# NiH‐Catalyzed Enantio‐ and Regioselective Hydroselenylation of Unactivated Alkenes

**DOI:** 10.1002/advs.77070

**Published:** 2026-08-11

**Authors:** Hyung‐Joon Kang, Jaehun Kim, Sungwoo Hong

**Affiliations:** ^1^ Center for Catalytic Hydrocarbon Functionalizations Institute for Basic Science (IBS) Daejeon Republic of Korea; ^2^ Department of Chemistry Korea Advanced Institute of Science and Technology (KAIST) Daejeon Republic of Korea

**Keywords:** alkenes, asymmetric synthesis, catalysis, chalcogen, hydroselenylation, nickel

## Abstract

Chiral alkyl selenides are valuable motifs in medicinal chemistry and redox‐active molecular design, yet enantioselective hydroselenylation of unactivated alkenes remains challenging due to limited control over regio‐ and enantioselectivity. Here, we report nickel‐hydride‐catalyzed enantio‐ and regioselective hydroselenylation of unactivated alkenes directed by native amide groups using readily available diselenides. The method accommodates both terminal and internal alkenes as well as both aryl and alkyl diselenides, exhibiting broad functional‐group tolerance and delivering structurally diverse chiral aliphatic selenides with high regio‐ and enantioselectivity under mild conditions. Mechanistic studies support enantiodetermining hydronickelation followed by single‐electron diselenide activation and selective capture of the selenium radical by the chiral alkyl–nickel intermediate. This work establishes a general platform for asymmetric C─Se bond formation through the integration of NiH catalysis and radical selenium transfer.

## Graphical abstract

Nickel‐hydride‐catalyzed enantio‐ and regioselective hydroselenylation of unactivated alkenes provides access to chiral organoselenium compounds. Enantiodetermining Ni–H insertion followed by single‐electron diselenide activation generates selenyl radical. The selenyl radical intercepts the chiral alkyl–Ni intermediate to forge C─Se bonds, while electrophilic trapping of the selenide anion prevents catalyst deactivation and sustains turnover.

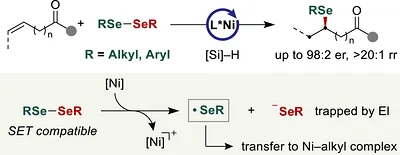

## 1 Introduction

Selenium occupies a distinctive position among the chalcogens due to its enhanced polarizability and redox flexibility relative to sulfur [1, 2, 3, 4]. These physicochemical features—facile oxidation, rapid reduction, and strong σ‐donor yet weak π‐bonding character—endow organoselenium compounds with unique reactivity profiles widely exploited in biology, medicinal chemistry, and catalysis (Figure 1A) [5, 6, 7, 8, 9, 10, 11, 12, 13, 14]. Incorporation of selenium into molecular frameworks can modulate lipophilicity, conformational bias, chalcogen‐bonding interactions, and redox responsiveness, often leading to altered biological activity compared to sulfur analogs [15, 16, 17, 18, 19]. Moreover, the three‐dimensional orientation of selenium substituents profoundly influences molecular recognition and binding affinity. Given that stereochemistry dictates both pharmacological activity and physicochemical properties in bioactive molecules [20, 21, 22, 23, 24, 25, 26], efficient access to enantioenriched organoselenium compounds is essential for exploiting selenium's unique attributes in drug discovery and chemical biology.

**FIGURE 1:**
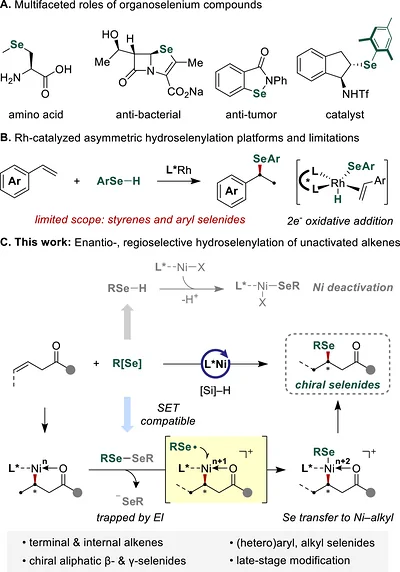
Development of NiH‐catalyzed enantioselective hydroselenylation.

Direct enantio‐ and regioselective hydroselenylation of simple alkenes represents an atom‐economical approach to access chiral organoselenium compounds. Catalytic asymmetric hydroselenylation has been most successfully achieved through Rh‐hydride catalysis, in which oxidative addition of aryl selenols initiates a classical two‐electron catalytic cycle (Figure 1B) [27]. This strategy has enabled efficient C─Se bond formation for electronically activated alkenes, particularly styrenes, establishing key precedents for enantioselective selenium incorporation. However, several limitations constrain this approach. Unactivated alkenes lacking electronic bias display little reactivity, coordinating functional groups interfere with catalyst control and regioselectivity, and the method is restricted to aryl selenol partners. These limitations necessitate alternative catalytic strategies capable of accommodating unactivated alkenes and diverse selenium sources.

To overcome these limitations, we pursued a mechanistically distinct strategy based on asymmetric NiH catalysis. Direct application of the Se─H oxidative addition manifold to nickel is problematic because selenols form stable Ni─Se complexes that suppress catalytic turnover. Indeed, well‐defined Ni─Se bonds in discrete nickel complexes display high thermodynamic stability, underscoring the strong binding affinity of selenium to nickel [28, 29, 30]. We therefore hypothesized that an effective NiH‐catalyzed hydroselenylation requires a selenium source that (i) engages in single‐electron activation rather than oxidative addition and (ii) minimizes premature catalyst deactivation through strong Ni─Se coordination. Diselenides fulfill these criteria. While the Engle group reported a single example in which a diselenide served as the electrophile in a racemic carboselenylation enabled by a bidentate directing group [31], asymmetric hydroselenylation using a native‐carbonyl‐directed NiH manifold remains unexplored. We envisioned that carbonyl‐directed hydronickelation of the alkene would generate a chiral alkyl–nickel intermediate capable of reducing the Se─Se bond via single‐electron transfer (SET). The resulting selenium radical would undergo selective capture by the Ni–alkyl species, forging the C─Se bond while the generated selenide anion is intercepted by an external electrophile. This design exploits the single‐electron reactivity characteristic of nickel while circumventing the two‐electron oxidative addition and minimizing off‐cycle Ni─Se formation. Herein, we report NiH‐catalyzed enantio‐ and regioselective hydroselenylation of unactivated linear alkenes using both aryl and alkyl diselenides (Figure 1C). Native amide directing groups enable precise hydronickelation control, delivering chiral alkyl selenides with excellent regio‐ and enantioselectivity under mild conditions. The transformation exhibits broad functional‐group tolerance and accommodates alkyl diselenides inaccessible via Rh‐based methods, significantly expanding the scope of asymmetric hydroselenylation.

## 2 Results and Discussion

### 2.1 Reaction Optimization

To realize this design principle, we sought to identify a selenium source compatible with single‐electron transfer (SET) processes under NiH catalysis [32, 33, 34, 35, 36, 37, 38, 39, 40, 41]. We initiated our investigation by evaluating various selenium‐transfer reagents for the asymmetric hydroselenylation of *N*‐phenylbut‐3‐enamide (**1a**) using NiBr_2_·glyme (10 mol%) and L* (12 mol%) in the presence of (MeO)_2_MeSiH, Na_2_CO_3_ (2.0 equiv), and the additive BnBr (1.0 equiv) and NaBF_4_ (1.0 equiv) in THF at 25°C (Table 1 and Tables S1–S5). Crucially, despite the presence of the trapping agent and salt additive—the latter of which is known to promote fluoride‐assisted silane activation and accelerate Ni─H formation [37, 38, 39, 40, 42, 43], conventional electrophilic selenium sources proved completely unreactive. Nucleophilic phenyl selenol (**[Se]‐1**) and traditional electrophilic selenyl halides (**[Se]‐2** and **[Se]‐3**) failed to deliver any of the desired product (Table 1, entries 1–3). This lack of reactivity underscores their fundamental incompatibility with the highly reducing NiH manifold. Selenols readily generate strongly coordinating selenolate species that form catalytically dead‐end off‐cycle Ni─Se species, while selenyl halides are prone to rapid, unproductive reduction or the formation of robust, stable Ni─Se/Ni─X coordinates under these basic, reducing conditions [44]. Similarly, phthalimide‐ and tosyl‐substituted selenium sources (**[Se]‐5** and **[Se]‐6**) were entirely unproductive under these fully optimized conditions (Table 1, entries 5 and 6). In stark contrast, diphenyl diselenide (**[Se]‐4**) emerged as a uniquely competent reagent, furnishing the desired β‐selenide **3a** in an excellent 91% yield with outstanding regioselectivity (18:1 rr) and enantiomeric ratio (97:3 er) (Table 1, entry 4). These outcomes clearly demonstrate that the unique SET‐active architecture of the diselenide framework is strictly required to engage with the active NiH catalyst and enable efficient catalytic turnover. This finding effectively reconciles the apparent limitation of atom economy; while a diselenide theoretically offers lower atom efficiency than selenyl halides, the latter is chemically inert within this catalytic system, making the diselenide framework an indispensable choice for successful hydroselenylation. To further probe the roles of the individual reaction components, a series of control experiments was conducted using diphenyl diselenide (**[Se]‐4**) as the standard selenium source. Omitting the benzyl bromide scavenger resulted in a dramatic collapse in efficiency, yielding only 11% of the product (Table 1, entry 7). This observation strongly supports our hypothesis that single‐electron reduction of the diselenide generates an equivalent of a nucleophilic selenolate anion (Se^−^) that poisons the nickel catalyst; the presence of benzyl bromide is therefore essential to cleanly intercept this transient byproduct as benzyl phenyl selenide and maintain catalyst viability. Similarly, replacing NaBF_4_ with simple fluoride salts such as NaF or CsF afforded **3a** in only 8% yield (Table 1, entry 8), corresponding to less than one turnover under the standard catalyst loading. These results support the importance of the weakly coordinating BF4^−^ counteranion in maintaining cationic nickel intermediates and enabling efficient catalyst regeneration. Reducing the catalyst loading to 5.0 mol% Ni and 6.0 mol% L* marginally decreased the yield to 87% (Table 1, entry 9). Substituting NiBr_2_·glyme for NiCl_2_·glyme or replacing (MeO)_2_MeSiH with Ph_2_SiH_2_ led to slightly diminished yields of 89% and 81%, respectively (Table 1, entries 10 and 11), while maintaining high stereoselectivities. Optimization was finalized by increasing the BnBr loading to 2.0 equivalents, which provided **3a** in an exceptional 95% isolated yield without compromising the regio‐ or enantioselectivity (17:1 rr, 97:3 er, Table 1, entry 12). The inclusion of Na_2_CO_3_ was found to be essential, as its omission led to severe suppression of the reaction, presumably by preventing protonation of transient selenium intermediates into inhibitory selenol species. Omitting either the nickel catalyst or the silane completely halted the reaction (Table 1, entry 13), confirming the necessity of the NiH system. Finally, replacing diphenyl diselenide with diphenyl disulfide afforded the corresponding hydrothiolation product in only 7% yield (Scheme S1). The diminished reactivity is consistent with the more negative reduction potential of PhSSPh and the greater strength of the S─S bond, identifying sulfur electrophiles as a current limitation of the catalytic system.

**TABLE 1:** Optimization of reaction conditions.^a^

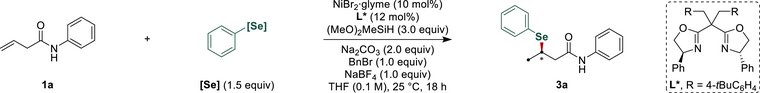					

entry	[Se]	Deviation from standard conditions	3a (%)	rr	er
1	**1**	none	—	—	—
2	**2**	none	—	—	—
3	**3**	none	—	—	—
4	**4**	none	91	18:1	97:3
5	**5**	none	—	—	—
6	**6**	none	—	—	—
7	**4**	without BnBr	11	15:1	94:6
8	**4**	NaF or CaF instead of NaBF_4_	8	12:1	92:8
9	**4**	Ni, L* (5.0, 6.0 mol%)	87	18:1	97:3
10	**4**	NiCl_2_ glyme	89	18:1	96:4
11	**4**	Ph_2_SiH_2_	81	16:1	96:4
12	**4**	BnBr (2.0 equiv)	95 (95)^b^	17:1	97:3
13	**4**	w/o catalyst or silane	—	—	—
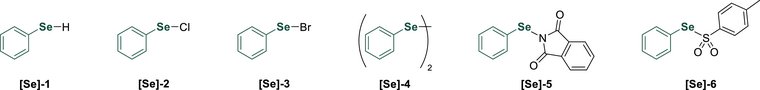					

^a^ Reactions were performed using **1a** (0.1 mmol), **[Se]** (1.5 equiv), NiBr_2_∙glyme (10 mol%), L* (12 mol%), (MeO)_2_MeSiH (3.0 equiv), Na_2_CO_3_ (2.0 equiv), BnBr (1.0 equiv) and NaBF_4_ (1.0 equiv) in THF (0.1 m, 1.0 mL) at 25°C under Ar for 18 h. Yields and regioisomeric ratios (rr) were determined by ^1^H NMR analysis of the crude mixture. Enantiomeric ratios (er) were determined by SFC analysis.

^b^ Isolated yield is shown in parentheses.

### 2.2 Substrate Scope Studies

Having established the optimized conditions, we investigated various alkene substrates to highlight the broad synthetic applicability of our hydroselenylation method (Table 2). Various terminal and internal alkenes bearing amides reacted smoothly, exhibiting excellent regio‐ and enantioselectivities, demonstrating the efficiency of this methodology. Our investigation began with terminal β,γ‐alkenes. Both electron‐donating (**3b** and **3c**) and electron‐withdrawing (**3d**−**3f**) groups at the para‐position of the N‐phenyl ring were well‐tolerated, yielding the desired products efficiently. A sterically more demanding ortho‐substituent (**3g**) was compatible with the reaction conditions, yielding the product in high efficiency. The *N*‐alkyl substituted enamide (**3h**) also showed high yield and stereoselectivity. Regardless of the starting alkene configuration (*Z* or *E*), *N*‐phenylhex‐3‐enamides underwent hydroselenylation to afford the corresponding products. The (*Z*)‐alkene exhibited superior reactivity (94% yield, 96:4 er), whereas the (*E*)‐alkene yielded **3i** in a slightly diminished yield (67%) while retaining excellent stereoselectivity (95:5 er). A substituent on the *N*‐phenyl group (**3j**) had minimal impact on the outcome, and both linear (**3k**) and cyclic (**3l**) alkyl‐substituted alkenes proved to be compatible with the optimized conditions. Specifically, terminal motifs such as cyclic alkyl (**3m**) and phenyl (**3n**) groups were successfully accommodated, delivering high yields and superior stereocontrol. Furthermore, the reaction could be extended to a γ,δ‐alkene (**3o**). Notably, the utility of this protocol was not limited to *N*‐aryl amides; the use of benzamide as an alternative directing group (**3p**) also proved successful, significantly broadening the synthetic scope of the method. To further demonstrate the synthetic utility, the protocol was applied to an L‐phenylalanine‐derived alkene, which successfully afforded the hydroselenylation product (**3q**) in good yield with respectable diastereoselectivity. Notably, an isoxepac, and indomethacin‐derived linear alkene also proved to be a viable substrate, delivering the corresponding selenylated products (**3r** and **3s**) with synthetically useful regio‐ and enantioselectivity. These results demonstrate that our protocol can be selectively and efficiently applied to complex molecules, highlighting its potential for the late‐stage functionalization of biorelevant compounds. With the versatility of the alkene component established, we turned to evaluating the breadth of the selenium coupling partner. Encouragingly, a structurally diverse array of aryl, heteroaryl, and alkyl diselenides proved to be competent substrates, delivering the corresponding β‐selenylated products in good to excellent yields with consistently high enantioselectivity. The electronic nature of the aryl substituents had minimal influence on the reaction outcome. Diaryl diselenides bearing electron‐donating (**4a** and **4e**) and electron‐withdrawing groups (**4b**–**4d**) at the para‐position were smoothly converted, affording the desired products with high efficiency and excellent enantiocontrol. Steric effects were likewise well tolerated: *ortho*‐substituted (**4f**), 2,3‐disubsituted (**4g**), and even 1,3,5‐trisubstituted aryl diselenides (**4h**) furnished the products in good to excellent yields without erosion of enantioselectivity. A meta‐substituted diselenide (**4i**) also participated readily, further underscoring the robustness of the transformation. The method extends beyond simple aryl systems. A representative heteroaryl diselenide smoothly underwent hydroselenylation to furnish the thiophene‐containing product **4j** with excellent enantioselectivity; the absolute configuration of **4j** was established as (*R*) by single‐crystal x‐ray diffraction analysis. Importantly, aliphatic diselenides were likewise viable substrates: alkyl diselenides bearing cycloalkyl (**4k**) and benzyl (**4l**) groups engaged smoothly under the standard conditions.

**TABLE 2:** Scope of amide substrates.^a^

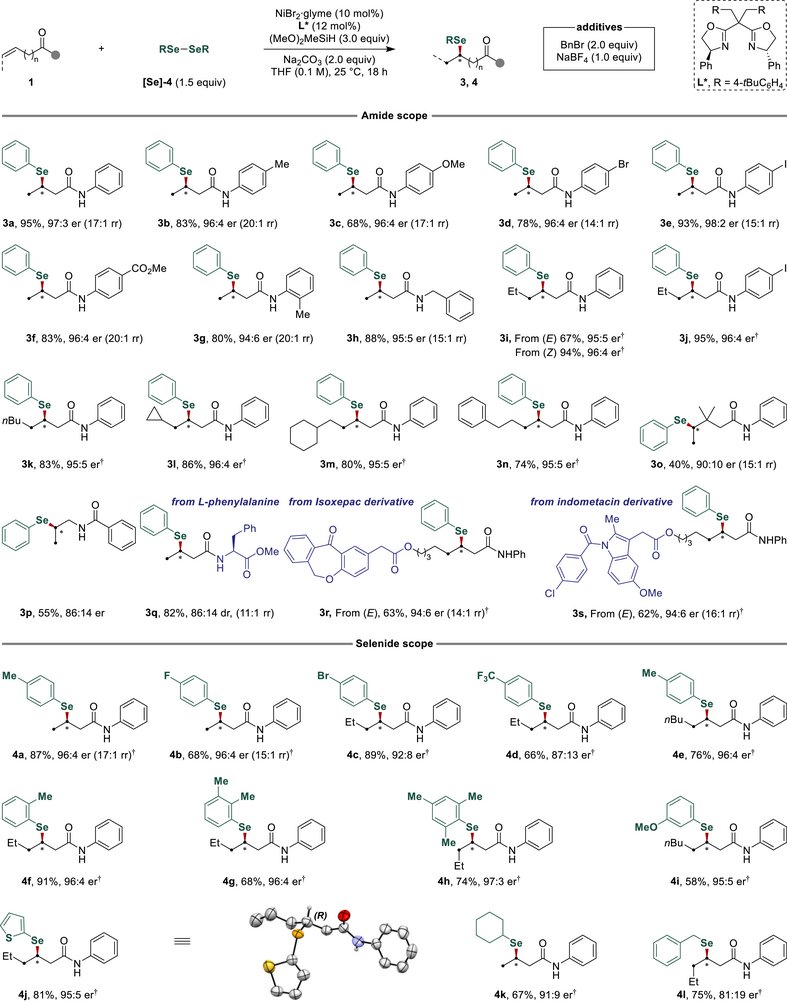

^a^ Reactions were performed using **1** (0.1 mmol), **[Se]‐4** (1.5 equiv), NiBr_2_∙glyme (10 mol%), L* (12 mol%), (MeO)_2_MeSiH (3.0 equiv), and Na_2_CO_3_ (2.0 equiv) in THF (0.1 m, 1.0 mL) at 25°C under Ar for 18 h. (*Z*)‐Alkenes were used if not denoted otherwise. Yields of isolated major products; regioisomeric ratios were determined by ^1^H NMR analysis of the crude mixture (>20:1 rr if not denoted otherwise). Enantiomeric ratios (er) were determined by SFC analysis.^†^NiCl_2_∙glyme was used instead of NiBr_2_∙glyme.

### 2.3 Mechanistic Investigations

To gain insight into the reaction mechanism, a series of control experiments was conducted (Figure 2). A deuterium‐labeling experiment employing Ph_2_SiD_2_ in place of (MeO)_2_MeSiH furnished ***d*‐3i** as a single diastereomer in 68% yield with complete deuterium incorporation (> 99% D) at the γ‐position and excellent enantioselectivity (Figure 2A). This result indicates that Ni─H migratory insertion is irreversible and constitutes the regio‐ and enantiodetermining step. A nonlinear effect study revealed a linear correlation between ligand and product enantiomeric excess (Figure 2B and Figure S1), supporting the involvement of a monomeric nickel species in the enantiodetermining step. A pronounced counter‐anion effect was observed (Figure 2C). Fluoride‐containing anions (BF_4_^−^, PF_6_^−^) significantly enhanced reaction efficiency, consistent with their role in facilitating Ni─H regeneration. In contrast, triflate failed to promote catalytic turnover, highlighting the critical role of fluoride‐derived counter‐anions in sustaining efficient NiH catalysis [36, 37, 38, 39, 42, 43]. We next investigated the proposed single‐electron activation of PhSe–SePh. Under standard conditions on 1.0 mmol scale, hydroselenylation afforded **3a** in comparable yield and selectivity. Notably, BnSePh was formed in 1.77 mmol (mass recovery relative to **1a**), supporting the generation of a nucleophilic selenium species during diselenide activation (Figure 2D). The excess BnSePh (> 1.5 equiv) likely arises from secondary pathways in which the initially generated selenyl radical is further converted to selenide anion via single‐electron reduction [45, 46, 47] or hydrogen‐atom transfer from the silane [48, 49], followed by deprotonation. Such processes would generate additional nucleophilic selenium species capable of benzylation. These results are consistent with the formation of selenium‐centered radical and nucleophilic selenium species, where BnBr traps nucleophilic selenium species to prevent off‐cycle Ni─Se coordination.

**FIGURE 2:**
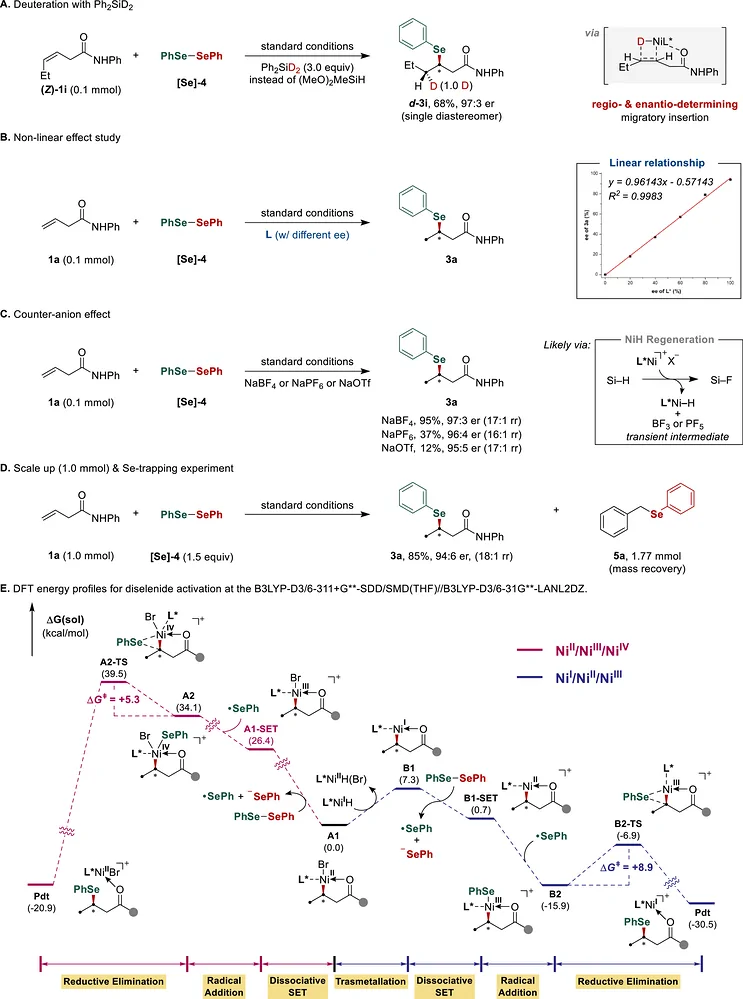
Mechanistic investigations. Please see Table S6 for the computed energy components for optimized structures.

Density functional theory (DFT) calculations were conducted to interrogate single‐electron activation of diphenyl diselenide and to assess the feasibility of competing nickel oxidation‐state manifolds (Figure 2E) [50, 51, 52, 53, 54, 55, 56, 57]. Starting from the post‐insertion alkyl–Ni^II^ intermediate (**A1**, 0.0 kcal/mol), single‐electron transfer along the Ni^II^/Ni^III^/Ni^IV^ pathway is highly disfavored, requiring a substantial free‐energy input (+26.4 kcal/mol, **A1‐SET**) and leading to a significantly destabilized intermediate (+34.1 kcal/mol, **A2**). These energetics render the high‐valent pathway unlikely under the mild reaction conditions. This conclusion is further supported by redox considerations: the calculated oxidation potential for **A1‐SET** (E^°^[Ni^II^/ Ni^III^] = −0.32 V vs. SCE), when paired with the experimental reduction potential of diphenyl diselenide (E_red_ = −0.85 V vs. SCE) [58], corresponds to a thermodynamically uphill electron‐transfer event (E_cell_ = −0.53 V). In contrast, the Ni^I^/Ni^II^/Ni^III^ manifold is both kinetically and thermodynamically viable. Formation of the alkyl–Ni^I^ species (**B1**) via transmetallation from **A1** proceeds with a modest free‐energy cost (+7.3 kcal/mol), after which single‐electron activation of the diselenide occurs with a negligible barrier (**B1‐SET**, +0.7 kcal/mol relative to **A1**), delivering a markedly stabilized alkyl–Ni^III^ intermediate (**B2**, −15.9 kcal/mol). The calculated oxidation potential for this step (E^°^[Ni^I^/Ni^II^] = +1.76 V vs. SCE) results in a strongly favorable electron‐transfer process when coupled with diselenide reduction (E_cell_ = +0.91 V), providing a clear thermodynamic driving force for SET. Subsequent C─Se bond‐forming reductive elimination proceeds through **B2‐TS** with a modest activation barrier (8.9 kcal/mol), consistent with a facile product‐forming step. These computational results provide compelling evidence that diselenide activation proceeds preferentially through the lower‐valent Ni^I^/Ni^II^/Ni^III^ redox manifold, in excellent agreement with the experimentally observed reactivity. While participation of a Ni^II^/Ni^III^/Ni^IV^ pathway cannot be categorically excluded, it is strongly disfavored both kinetically and thermodynamically.

Based on the control experiments, a plausible catalytic cycle for the nickel‐catalyzed asymmetric hydroselenylation is proposed in Figure 3. The cycle is initiated by the generation of the active species L*Ni^II^–H and L*Ni^I^–H from the reaction of the nickel precursor with hydrosilane. The more Lewis acidic L*Ni^II^–H preferentially coordinates to the carbonyl directing group of alkene **1a**, enabling regio‐ and enantioselective hydronickellation to give the alkyl–Ni^II^–H intermediate **A1**. Transmetalation between **A1** and the Ni^I^−H species then generates the alkyl–Ni^I^−H intermediate **B1** and regenerates L*Ni^II^–H. **B1** undergoes single‐electron transfer (SET) to diphenyl diselenide (PhSe–SePh), producing a selenyl radical (•SePh), a nucleophilic selenolate anion (PhSe^−^), and the electrophilic cationic alkyl– Ni^II^ intermediate **B1‐SET**. To prevent catalyst poisoning by the strongly coordinating selenolate, PhSe^−^ is rapidly intercepted by benzyl bromide to form BnSePh as a sacrificial byproduct, while radical recombination between **B1‐SET** and •SePh affords the high‐valent alkyl–Ni^III^–selenide intermediate **B2**. Reductive elimination from **B2** delivers the β‐selenide product and a cationic Ni^I^ complex, and the cycle is closed by regeneration of the active Ni─H species through reaction with hydrosilane in the presence of fluoride.

**FIGURE 3:**
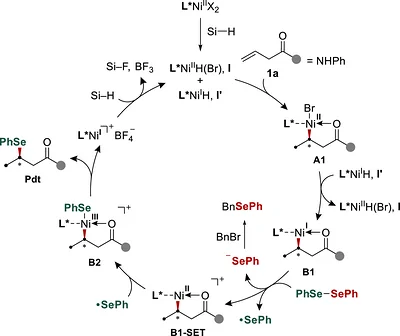
Plausible catalytic cycle.

## 3 Conclusion

We report a nickel‐hydride‐catalyzed enantioselective hydroselenylation of unactivated alkenes directed by native carbonyl groups. The method accommodates terminal and internal alkenes as well as diverse diaryl, heteroaryl, and dialkyl diselenides, furnishing selenylated alkyl products with high regio‐ and enantioselectivity. Mechanistic studies support irreversible enantiodetermining Ni─H migratory insertion followed by single‐electron reduction of the diselenide and selective selenium transfer, with benzyl bromide preventing off‐cycle Ni─Se coordination. By integrating enantioselective Ni─H insertion with controlled selenium transfer, this work establishes a mechanistically distinct platform for asymmetric C─Se bond formation and expands access to chiral organoselenium compounds.

## Author Contributions

**Hyung‐Joon Kang**: conceptualization, methodology, validation, visualization, Writing – original draft, Writing – review and editing, formal analysis, data curation, resources, software. **Jaehun Kim**: methodology, investigation, validation, visualization, writing – original draft, writing – review and editing, formal analysis. **Sungwoo Hong**: conceptualization, investigation, writing – original draft, funding acquisition, writing – review and editing, visualization, project administration, resources, supervision.

## Conflicts of Interest

The authors declare no conflicts of interest.

## Supporting information




**Supporting File**: advs77070‐sup‐0001‐SuppMat.docx.

## Data Availability

Deposition number 2526640 (for **4j**) contains the supplementary crystallographic data for this paper. These data are provided free of charge by the joint Cambridge Crystallographic Data Centre and Fachinformationszentrum Karlsruhe Access Structures service.
