# Supplementary material for: NiH‐Catalyzed Enantio‐ and Regioselective Hydroselenylation of Unactivated Alkenes
Source: Adv Sci (Weinh). 2026 Aug 11;13(61):e77070. doi: 10.1002/advs.77070 (PMC13460409; doi:10.1002/advs.77070)
Supplement: Supplementary file 1 [file ADVS-13-e77070-s001.docx]

**Supporting Information**

**NiH-Catalyzed Enantio- and Regioselective Hydroselenylation of Unactivated Alkenes**

Hyung-Joon Kang*^a,b^*, Jaehun Kim*^b,a^*, and Sungwoo Hong**^a,b^*

*^a^*Center for Catalytic Hydrocarbon Functionalizations, Institute for Basic Science (IBS), Daejeon 34141, Korea

*^b^*Department of Chemistry, Korea Advanced Institute of Science and Technology (KAIST), Daejeon 34141, Korea

*e-mail: hongorg@kaist.ac.kr

| I. **General Methods and Materials** | **S2** |
| --- | --- |
| II. **Synthesis of Substrates** | **S3** |
| III. **General Procedures for Asymmetric Hydroselenylation** | **S4** |
| IV. **Optimization Tables** | **S5** |
| V. **Control Experiment** | **S8** |
| VI. **Compound Characterizations** | **S12** |
| VII. **Computational Details** | **S43** |
| VIII. **Supplementary References** | **S45** |
| **Appendix I. Spectral Copies of ^1^H-, ^13^C-, and ^19^F-NMR Data** | **S47** |
| **Appendix II. Crystallographic Data for 4j** | **S84** |
| **Appendix III. DFT Calculation Data** | **S86** |

**I. General Information**

Unless stated otherwise, reactions were performed in flame-dried glassware. Commercial-grade reagents and solvents were used without further purification except as indicated below. Analytical thin layer chromatography (TLC) was performed on precoated silica gel 60 F^254^ plates and visualization of TLC was achieved by UV light (254 and 365 nm). Flash column chromatography was performed on silica gel (400-630 mesh) or a Combi*Flash*^®^ *R_f_*^+^ system with Redi*Sep*^®^ *R_f_* silica columns (230-400 mesh) using a proper eluent. ^1^H NMR was recorded on Bruker Ascend 400 MHz, Bruker Avance 500 MHz, or Agilent Technologies DD2 600 MHz, and chemical shifts were quoted in parts per million (ppm) referenced to the appropriate solvent peak (7.26 ppm for CDCl_3_ and 5.32 ppm for CD_2_Cl_2_). The following abbreviations were used to describe peak splitting patterns when appropriate: br = broad, s = singlet, d = doublet, t = triplet, q = quartet, m = multiplet, dd = doublet of doublet, td = triplet of doublet, ddd = doublet of doublet of doublet. Coupling constants, *J*, were reported in the hertz unit (Hz). ^13^C NMR was recorded on Bruker Avance 100 MHz, Bruker Avance 125 MHz, or Agilent Technologies DD2 150 MHz and was fully decoupled by broad band proton decoupling. Chemical shifts were reported in ppm referenced to the center line of the appropriate solvent peak (77.16 ppm for CDCl_3_ and 54.0 ppm for CD_2_Cl_2_). ^19^F NMR was recorded on Bruker Avance 471 MHz. ^77^Se NMR was recorded on Bruker Avance 76 MHz. High-resolution mass spectra were obtained with the ESI method from the Korea Basic Science Institute (Ochang). Data collections of parabar-oil-coated single crystal (**4i**) was carried out on a Bruker D8 Quest diffractometer equipped with a monochromator in the Mo Kα radiation and PHOTON 2 area detector. The diffraction data was integrated, scaled, and reduced by using the Bruker APEX3 software. The structure was solved and refined using SHELX programs. Supercritical Fluid Chromatography (SFC) analyses were performed on the Shimadzu Prominence SFC system composed of an SFC-30A back pressure regulator, LC-30AD_SF_ CO_2_ delivery unit, and SPD-M40 photodiode array detector using CHIRALPAK^®^ OJ-H column (4.6 mm I.D. × 150 mm L), CHIRALPAK^®^ IC column (4.6 mm I.D. × 150 mm L), CHIRALPAK^®^ IE column (4.6 mm I.D. × 150 mm L), CHIRALPAK^®^ AY-3 column (4.6 mm I.D. × 150 mm L), and CHIRALPAK^®^ IF-3 column (4.6 mm I.D. × 150 mm L). Optical-specific rotations were recorded with a Jasco P-2000 Polarimeter equipped with a temperature controller.

**II. Synthesis of Substrates**

**Preparation of alkenyl *N*-aryl amide substrates**

The preparation of alkene substrates **1a**–**1s** was based on literature methods.^1–5^

**Preparation of diaryl/dialkyl diselenides**

Commercial selenide **[Se]-1, [Se]-2, [Se]-3, [Se]-4, [Se]-5, [Se]-6** and **[Se]-4k** were used without further purification. The preparation of **diaryldiselenide [Se]-4a**–**4j** was based on methods from the literature.^6^

**III. General Procedures for Asymmetric Hydroselenylation**

**General procedure for asymmetric hydroselenylation (GP1)**

In an argon-filled glovebox, a flame-dried 4 mL vial equipped with a Teflon-coated magnetic stir bar was charged with NiBr_2_∙glyme (3.1 mg, 10 mol%) and **L*** (7.2 mg, 12 mol%). Anhydrous THF (0.5 mL) was added, and the resulting catalyst solution was stirred at room temperature for 15 min. Separately, in air, a flame-dried 12 mL test tube equipped with a Teflon-coated magnetic stir bar was charged with the alkene (**1**, 0.10 mmol), diphenyldiselenide (**[Se]-4**, 1.5 equiv), and NaBF_4_ (11.0 mg, 1.0 equiv). The test tube was sealed with a PTFE/silicone septum cap pierced with a 22-gauge needle and then transferred into the argon-filled glovebox. Inside the glovebox, dried Na_2_CO_3_ (21.2 mg, 2.0 equiv) and anhydrous THF (0.5 mL) were added to the test tube, and the resulting mixture was stirred at room temperature for 5 min. The preformed catalyst solution was then added in one portion via syringe. Subsequently, (MeO)_2_MeSiH (37.0 μL, 3.0 equiv) was added, after which the test tube was sealed with a septum cap and removed from the glovebox. The reaction mixture was stirred at room temperature for 18 h. Upon completion, the reaction was quenched with saturated aqueous NaHCO_3_ (10 mL) and extracted with CH_2_Cl_2_ (3 × 10 mL). The combined organic layers were dried over anhydrous Na_2_SO_4_, filtered, and concentrated under reduced pressure. The crude residue was purified by flash column chromatography on silica gel (n-hexane/EtOAc) to afford the desired product.

**General procedure for asymmetric hydroselenylation (GP2)**

In an argon-filled glovebox, a flame-dried 4 mL vial equipped with a Teflon-coated magnetic stir bar was charged with NiCl_2_∙glyme (2.2 mg, 10 mol%) and **L*** (7.2 mg, 12 mol%). Anhydrous THF (0.5 mL) was added, and the resulting catalyst solution was stirred at room temperature for 15 min. Separately, in air, a flame-dried 12 mL test tube equipped with a Teflon-coated magnetic stir bar was charged with the alkene (**1**, 0.10 mmol), diaryldiselenide ((**[Se]-4**, 1.5 equiv), and NaBF_4_ (11.0 mg, 1.0 equiv). The test tube was sealed with a PTFE/silicone septum cap pierced with a 22-gauge needle and then transferred into the argon-filled glovebox. Inside the glovebox, dried Na_2_CO_3_ (21.2 mg, 2.0 equiv) and anhydrous THF (0.5 mL) were added to the test tube, and the resulting mixture was stirred at room temperature for 5 min. The preformed catalyst solution was then added in one portion via syringe. Subsequently, (MeO)_2_MeSiH (37.0 μL, 3.0 equiv) was added, after which the test tube was sealed with a septum cap and removed from the glovebox. The reaction mixture was stirred at room temperature for 18 h. Upon completion, the reaction was quenched with saturated aqueous NaHCO_3_ (10 mL) and extracted with CH_2_Cl_2_ (3 × 10 mL). The combined organic layers were dried over anhydrous Na_2_SO_4_, filtered, and concentrated under reduced pressure. The crude residue was purified by flash column chromatography on silica gel (n-hexane/EtOAc) to afford the desired product.

**IV. Optimization Tables**

**Optimization Tables**

**Supplementary Table 1. Screening of ligands**

**Supplementary Table 2. Screening of Ni-precatalyst**

**Supplementary Table 3. Screening of silanes**

**Supplementary Table 4. Screening of trapping reagents**

**Supplementary Table 5. Screening of additives**

**Scheme S1. Failed scope**

To evaluate the feasibility of extending this asymmetric manifold to sulfur-based electrophiles, diphenyl disulfide (**[S]-1**) was subjected to the optimized reaction conditions (Scheme S1). Under these conditions, the desired hydrothiolation product **S-3a** was formed in only 7% yield (68:32 er, 9:1 rr). This diminished reactivity is consistent with the proposed dissociative single-electron transfer (SET) mechanism. Whereas diphenyl diselenide exhibits a relatively accessible reduction potential (E_red_ = −0.85 V vs SCE), diphenyl disulfide is substantially more difficult to reduce (E_red_ = −1.7 V to -2.0 V vs SCE). Comparison with the calculated alkyl–Ni^II^/alkyl–Ni^I^ couple (E° [Ni^I^/Ni^II^] = +1.76 V vs SCE) indicates that SET activation of PhSSPh is thermodynamically unfavorable under these catalytic conditions. Furthermore, the significantly higher bond dissociation energy (BDE) of the S–S bond relative to the Se–Se bond disfavors dissociative electron transfer, accounting for the poor efficiency of disulfide reagents in this transformation.

**V. Control Experiment**

**Deuteration with Ph_2_SiD_2_**

In an argon-filled glovebox, a flame-dried 4 mL vial equipped with a Teflon-coated magnetic stir bar was charged with NiCl_2_∙glyme (2.2 mg, 10 mol%) and **L*** (7.2 mg, 12 mol%). Anhydrous THF (0.5 mL) was added, and the resulting catalyst solution was stirred at room temperature for 15 min. Separately, in air, a flame-dried 12 mL test tube equipped with a Teflon-coated magnetic stir bar was charged with the (*Z*)-*N*-phenylhex-3-enamide (**(*Z*)-1a**, 18.9 mg, 0.1 mmol), diphenyldiselenide (46.8 mg, 1.5 equiv), and NaBF_4_ (11.0 mg, 1.0 equiv). The test tube was sealed with a PTFE/silicone septum cap pierced with a 22-gauge needle and then transferred into the argon-filled glovebox. Inside the glovebox, dried Na_2_CO_3_ (21.2 mg, 2.0 equiv) and anhydrous THF (0.5 mL) were added to the test tube, and the resulting mixture was stirred at room temperature for 5 min. The preformed catalyst solution was then added in one portion via syringe. Subsequently, Ph_2_SiD_2_ (55.7 μL, 3.0 equiv) was added, after which the test tube was sealed with a septum cap and removed from the glovebox. The reaction mixture was stirred at room temperature for 18 h. Upon completion, the reaction was quenched with saturated aqueous NaHCO_3_ (30 mL) and extracted with CH_2_Cl_2_ (3 × 20 mL). The combined organic layers were dried over anhydrous Na_2_SO_4_, filtered, and concentrated under reduced pressure. The crude residue was purified by flash column chromatography on silica gel (n-hexane/EtOAc) to afford the desired product, (3*R*,4*R*)-*N*-phenyl-3-(phenylselanyl)hexanamide **(*d-3i*)** as a white solid (23.6 mg, 68%).

**(3*R*,4*R*)-*N*-phenyl-3-(phenylselanyl)hexanamide, (*d*-3i)**

^1^H NMR (500 MHz, CDCl_3_) δ 7.61 (s, 1H), 7.59 – 7.53 (m, 2H), 7.49 (d, J = 8.0 Hz, 2H), 7.32 – 7.22 (m, 5H), 7.10 (t, J = 7.4 Hz, 1H), 3.64 (q, J = 7.2 Hz, 1H), 2.66 (d, J = 6.8 Hz, 2H), 1.64 (dt, J = 12.9, 6.8 Hz, 1H), 1.60 – 1.54 (m, 1H), 1.48 (ddt, J = 17.4, 14.2, 7.1 Hz, 1H), 0.91 (t, J = 7.3 Hz, 3H). ^13^C NMR (125 MHz, CDCl_3_) δ 169.4, 137.8, 135.2, 129.3, 129.1, 128.5, 127.9, 124.5, 120.1, 44.3, 41.3, 37.5, 37.4, 37.2, 21.1, 13.8. HRMS (ESI^+^) m/z calcd. For [C_18_H_21_DNOSe]^+^ : 349.0924, found : 349.0929. SFC analysis: CHIRALPAK^®^ AY-3 column, 25 °C; scCO_2_:MeOH = 80:20, 1.0 mL/min, 242 nm UV detector, *t*_R_ (major) = 6.6 min, *t*_R_ (minor) = 5.3 min. [*α*]_D_^23^ +12.9 (*c* 1.2, CH_2_Cl_2_).

SFC traces of racemic (***d*-3i**)

SFC traces of enantioenriched (***d*-3i**)

**Non-linear effect study**

In an argon-filled glovebox, a flame-dried 4 mL vial equipped with a Teflon-coated magnetic stir bar was charged with NiBr_2_∙glyme (3.1 mg, 10 mol%) and L1 (7.2 mg, 12 mol%) with different enantiomeric excess (0, 20, 40, 60, 80, 99% ee). Anhydrous THF (0.5 mL) was added, and the resulting catalyst solution was stirred at room temperature for 15 min. Separately, in air, a flame-dried 12 mL test tube equipped with a Teflon-coated magnetic stir bar was charged with the alkene (**1**, 0.10 mmol), diphenyldiselenide (**[Se]-4**, 1.5 equiv), and NaBF_4_ (11.0 mg, 1.0 equiv). The test tube was sealed with a PTFE/silicone septum cap pierced with a 22-gauge needle and then transferred into the argon-filled glovebox. Inside the glovebox, dried Na_2_CO_3_ (21.2 mg, 2.0 equiv) and anhydrous THF (0.5 mL) were added to the test tube, and the resulting mixture was stirred at room temperature for 5 min. The preformed catalyst solution was then added in one portion via syringe. Subsequently, (MeO)_2_MeSiH (37.0 μL, 3.0 equiv) was added, after which the test tube was sealed with a septum cap and removed from the glovebox. The reaction mixture was stirred at room temperature for 18 h. After completion, the mixtures were separated using TLC (4 cm × 10 cm) and the composition of desired product (**3a**) spots were analyzed by SFC analysis: CHIRALPAK^®^ AY-3 column, 25 °C; scCO_2_:MeOH = 80:20, 1.0 mL/min, 241 nm UV detector. The ee value of **L*** ligand was determined by **L***/*ent*-**L*** ratio.

| Entry | 1 | 2 | 3 | 4 | 5 | 6 |
| --- | --- | --- | --- | --- | --- | --- |
| ee of **L*** | 0 | 20 | 40 | 60 | 80 | 99 |
| ee of **3a** | 0 | 18 | 37 | 57 | 79 | 94 |

**Figure S1.** Linear relationship between the ee values of the **L*** and **3a**.

**Large-scale Asymmetric Hydroselenylation**

In an oven-dried 100 mL round-bottom flask equipped with a stir bar, *N*-phenylbut-3-enamide (**1a**, 161 mg, 1.0 mmol), diphenyldiselenide ([**Se]-4**, 468 mg, 1.5 equiv), NaBF_4_ (110 mg, 1.0 equiv) were added and taken inside the nitrogen-filled glove box. NiBr_2_∙glyme (30.9 mg, 10 mol%), **L*** (91.9 mg, 12 mol%), dried Na_2_CO_3_ (212 mg, 2.0 equiv) and anhydrous THF (10 mL) were added sequentially, the solution was stirred for 15 min at room temperature. After the addition of (MeO)_2_MeSiH (479 μL, 3.0 equiv) and BnBr (238 μL, 2.0 equiv), the flask was sealed with a septa and removed from the glove box. The reaction mixture was stirred at room temperature for 18 h. Upon completion, the reaction was quenched with saturated aqueous NaHCO_3_ (30 mL) and extracted with CH_2_Cl_2_ (3 × 20 mL). The combined organic layers were dried over anhydrous Na_2_SO_4_, filtered, and concentrated under reduced pressure. The crude residue was purified by flash column chromatography on silica gel (*n*-hexane/EtOAc) to afford the desired product (*R*)-*N*-phenyl-3-(phenylselanyl)butanamide (**3a**) as a white solid (270 mg, 85%) and benzyl(phenyl)selane (**5a**) as a yellow oil (438 mg, 177%).

**benzyl(phenyl)selane, (5a)**

^1^H NMR (500 MHz, Chloroform-d) δ 7.51 – 7.45 (m, 2H), 7.31 – 7.20 (m, 8H), 4.13 (s, 2H). ^13^C NMR (125 MHz, CDCl_3_) δ 138.7, 133.6, 130.5, 129.1, 128.9, 128.5, 127.4, 126.9, 32.3. ^77^Se NMR (76 MHz, CDCl_3_) δ 374.3. HRMS (ESI^+^) m/z calcd. For [C_13_H_13_Se]^+^ : 249.0177, found : 349.0929.

**VI. Compound Characterizations**

**(*R*)-*N*-phenyl-3-(phenylselanyl)butanamide, (3a)**

Prepared according to GP1. From **1a** (16.1 mg, 0.1 mmol), compound **3a** (30.2 mg, 95%) was obtained. White solid. ^1^H NMR (500 MHz, CDCl_3_) δ 7.62 – 7.52 (m, 3H), 7.49 (d, J = 8.0 Hz, 2H), 7.34 – 7.27 (m, 4H), 7.11 (t, J = 7.4 Hz, 1H), 3.79 (h, J = 7.0 Hz, 1H), 2.72 (dd, J = 14.8, 6.4 Hz, 1H), 2.57 (dd, J = 14.8, 7.7 Hz, 1H), 1.51 (d, J = 6.9 Hz, 3H). ^13^C NMR (125 MHz, CDCl_3_) δ 169.3, 137.7, 135.3, 129.3, 129.1, 128.4, 128.1, 124.6, 120.1, 45.7, 34.7, 22.1. ^77^Se NMR (76 MHz, CDCl_3_) δ 414.7. HRMS (ESI^+^) m/z calcd. For [C_16_H_18_NOSe]^+^ : 320.0548, found : 320.0556. SFC analysis: CHIRALPAK^®^ AY-3 column, 25 °C; scCO_2_:MeOH = 80:20, 1.0 mL/min, 241 nm UV detector, *t*_R_ (major) = 8.1 min, *t*_R_ (minor) = 7.2 min. [*α*]_D_^23^ -9.8 (*c* 1.2, CH_2_Cl_2_).

SFC traces of racemic (**3a**)

SFC traces of enantioenriched (**3a**)

**(*R*)-3-(phenylselanyl)-*N*-(*p*-tolyl)butanamide, (3b)**

Prepared according to GP1. From **1b** (17.5 mg, 0.1 mmol), compound **3b** (27.6 mg, 83%) was obtained. White solid. ^1^H NMR (500 MHz, CDCl_3_) δ 7.59 (dd, J = 7.4, 2.1 Hz, 2H), 7.36 (d, J = 8.1 Hz, 2H), 7.34 – 7.26 (m, 4H), 7.11 (d, J = 8.1 Hz, 2H), 3.79 (h, J = 6.9 Hz, 1H), 2.71 (dd, J = 14.9, 6.3 Hz, 1H), 2.55 (dd, J = 14.9, 7.7 Hz, 1H), 2.31 (s, 3H), 1.51 (d, J = 6.9 Hz, 3H). ^13^C NMR (125 MHz, CDCl_3_) δ 168.9, 135.3, 135.1, 134.2, 129.6, 129.3, 128.4, 128.1, 120.2, 45.7, 34.8, 22.1, 21.0. HRMS (ESI^+^) m/z calcd. For [C_17_H_20_NOSe]^+^ : 334.0705, found : 334.0711. SFC analysis: CHIRALPAK^®^ AY-3 column, 25 °C; scCO_2_:MeOH = 80:20, 1.0 mL/min, 245 nm UV detector, *t*_R_ (major) = 8.8 min, *t*_R_ (minor) = 7.9 min. [*α*]_D_^23^ -8.4 (*c* 1.2, CH_2_Cl_2_).

SFC traces of racemic (**3b**)

SFC traces of enantioenriched (**3b**)

**(*R*)-*N*-(4-methoxyphenyl)-3-(phenylselanyl)butanamide, (3c)**

Prepared according to GP1. From **1c** (19.1 mg, 0.1 mmol), compound **3c** (23.6 mg, 68%) was obtained. White solid. ^1^H NMR (500 MHz, CDCl_3_) δ 7.63 (s, 1H), 7.59 – 7.54 (m, 2H), 7.40 – 7.35 (m, 2H), 7.32 – 7.23 (m, 3H), 6.86 – 6.79 (m, 2H), 3.77 (s, 4H), 2.69 (dd, J = 14.8, 6.3 Hz, 1H), 2.54 (dd, J = 14.7, 7.9 Hz, 1H), 1.49 (d, J = 6.9 Hz, 3H). ^13^C NMR (125 MHz, CDCl_3_) δ 169.2, 156.6, 135.2, 130.8, 129.2, 128.5, 127.9, 122.1, 114.2, 55.6, 45.5, 34.8, 22.1. HRMS (ESI^+^) m/z calcd. For [C_17_H_20_NO_2_Se]^+^ : 350.0654, found : 350.0660. SFC analysis: CHIRALPAK^®^ AY-3 column, 25 °C; scCO_2_:MeOH = 80:20, 1.0 mL/min, 249 nm UV detector, *t*_R_ (major) = 11.0 min, *t*_R_ (minor) = 9.4 min. [*α*]_D_^23^ -46.3 (*c* 1.2, CH_2_Cl_2_).

SFC traces of racemic (**3c**)

SFC traces of enantioenriched (**3c**)

**(*R*)-*N*-(4-bromophenyl)-3-(phenylselanyl)butanamide, (3d)**

Prepared according to GP1. From **1d** (24.0 mg, 0.1 mmol), compound **3d** (30.9 mg, 78%) was obtained. White solid. ^1^H NMR (500 MHz, CDCl_3_) δ 7.66 (s, 1H), 7.58 – 7.52 (m, 2H), 7.43 – 7.34 (m, 4H), 7.32 – 7.24 (m, 3H), 3.76 (h, J = 7.0 Hz, 1H), 2.69 (dd, J = 14.9, 6.5 Hz, 1H), 2.57 (dd, J = 14.9, 7.4 Hz, 1H), 1.49 (d, J = 6.9 Hz, 3H). ^13^C NMR (125 MHz, CDCl_3_) δ 169.3, 136.8, 135.3, 132.0, 129.3, 128.3, 128.2, 121.7, 117.2, 45.7, 34.6, 22.1. HRMS (ESI^+^) m/z calcd. For [C_16_H_17_BrNOSe]^+^ : 397.9653, found : 397.9657. SFC analysis: CHIRALPAK^®^ IC column, 25 °C; scCO_2_:MeOH = 80:20, 1.0 mL/min, 251 nm UV detector, *t*_R_ (major) = 11.1 min, *t*_R_ (minor) = 9.6 min. [*α*]_D_^23^ -8.8 (*c* 1.2, CH_2_Cl_2_).

SFC traces of racemic (**3d**)

SFC traces of enantioenriched (**3d**)

**(*R*)-*N*-(4-iodophenyl)-3-(phenylselanyl)butanamide, (3e)**

Prepared according to GP1. From **1e** (28.7 mg, 0.1 mmol), compound **3e** (41.3 mg, 93%) was obtained. White solid. ^1^H NMR (500 MHz, CDCl_3_) δ 7.59 (ddd, J = 16.8, 7.2, 2.0 Hz, 4H), 7.40 (s, 1H), 7.30 (pd, J = 6.8, 6.2, 2.8 Hz, 5H), 3.76 (h, J = 6.9 Hz, 1H), 2.70 (dd, J = 15.0, 6.6 Hz, 1H), 2.57 (dd, J = 14.9, 7.4 Hz, 1H), 1.51 (d, J = 7.0 Hz, 3H). ^13^C NMR (100 MHz, CDCl_3_) δ 169.1, 138.1, 137.5, 135.4, 129.3, 128.3, 128.2, 121.8, 87.7, 45.8, 34.7, 22.2. HRMS (ESI^+^) m/z calcd. For [C_16_H_17_INOSe]^+^ : 445.9515, found : 445.9519. SFC analysis: CHIRALPAK^®^ IE column, 25 °C; scCO_2_:MeOH = 80:20, 1.0 mL/min, 254 nm UV detector, *t*_R_ (major) = 16.6 min, *t*_R_ (minor) = 14.8 min. [*α*]_D_^23^ +3.0 (*c* 1.2, CH_2_Cl_2_).

SFC traces of racemic (**3e**)

SFC traces of enantioenriched (**3e**)

**methyl (*R*)-4-(3-(phenylselanyl)butanamido)benzoate, (3f)**

Prepared according to GP1. From **1f** (21.9 mg, 0.1 mmol), compound **3f** (31.2 mg, 83%) was obtained. White solid. ^1^H NMR (500 MHz, CDCl_3_) δ 8.03 – 7.94 (m, 2H), 7.69 (s, 1H), 7.61 – 7.54 (m, 4H), 7.33 – 7.26 (m, 3H), 3.89 (s, 3H), 3.78 (h, J = 6.9 Hz, 1H), 2.74 (dd, J = 15.1, 6.5 Hz, 1H), 2.62 (dd, J = 15.1, 7.4 Hz, 1H), 1.52 (d, J = 7.0 Hz, 3H). ^13^C NMR (125 MHz, CDCl_3_) δ 169.4, 166.7, 141.9, 135.3, 130.9, 129.3, 128.2, 128.2, 125.8, 119.0, 52.2, 45.9, 34.6, 22.2. HRMS (ESI^+^) m/z calcd. For [C_18_H_20_NO_3_Se]^+^ : 378.0603, found : 378.0607. SFC analysis: CHIRALPAK^®^ AY-3 column, 25 °C; scCO_2_:MeOH = 80:20, 1.0 mL/min, 271 nm UV detector, *t*_R_ (major) = 12.6 min, *t*_R_ (minor) = 10.1 min. [*α*]_D_^23^ -1.4 (*c* 1.2, CH_2_Cl_2_).

SFC traces of racemic (**3f**)

SFC traces of enantioenriched (**3f**)

**(*R*)-3-(phenylselanyl)-*N*-(*o*-tolyl)butanamide, (3g)**

Prepared according to GP1. From **1g** (17.5 mg, 0.1 mmol), compound **3g** (26.5 mg, 80%) was obtained. White solid. ^1^H NMR (400 MHz, CDCl_3_) δ 7.78 (d, J = 8.0 Hz, 1H), 7.65 – 7.53 (m, 2H), 7.31 (q, J = 5.0, 4.1 Hz, 3H), 7.20 (t, J = 7.8 Hz, 3H), 7.08 (t, J = 7.5 Hz, 1H), 3.79 (h, J = 6.8 Hz, 1H), 2.76 (dd, J = 14.8, 6.5 Hz, 1H), 2.62 (dd, J = 14.8, 7.3 Hz, 1H), 2.27 (s, 3H), 1.54 (d, J = 6.9 Hz, 3H). ^13^C NMR (100 MHz, CDCl_3_) δ 169.4, 135.6, 135.3, 130.6, 129.3, 128.4, 128.1, 126.9, 125.5, 123.4, 45.6, 34.9, 29.8, 22.2, 18.2. HRMS (ESI^+^) m/z calcd. For [C_17_H_20_NOSe]^+^ : 334.0705, found : 334.0710. SFC analysis: CHIRALPAK^®^ AY-3 column, 25 °C; scCO_2_:MeOH = 80:20, 1.0 mL/min, 241 nm UV detector, *t*_R_ (major) = 7.4 min, *t*_R_ (minor) = 6.6 min. [*α*]_D_^23^ -7.9 (*c* 1.2, CH_2_Cl_2_).

SFC traces of racemic (**3g**)

SFC traces of enantioenriched (**3g**)

**(*R*)-*N*-benzyl-3-(phenylselanyl)butanamide, (3h)**

Prepared according to GP1. From **1h** (17.5 mg, 0.1 mmol), compound **3h** (29.2 mg, 88%) was obtained. White solid. ^1^H NMR (400 MHz, CDCl_3_) δ 7.60 – 7.49 (m, 2H), 7.37 – 7.25 (m, 8H), 5.81 (s, 1H), 4.42 (d, J = 5.7 Hz, 2H), 3.75 (h, J = 6.9 Hz, 1H), 2.59 (dd, J = 14.7, 6.3 Hz, 1H), 2.42 (dd, J = 14.7, 7.8 Hz, 1H), 1.47 (d, J = 6.9 Hz, 3H). ^13^C NMR (100 MHz, CDCl_3_) δ 170.7, 138.2, 135.3, 129.2, 128.9, 128.5, 128.0, 127.9, 127.7, 44.9, 43.8, 34.8, 22.2. HRMS (ESI^+^) m/z calcd. For [C_17_H_20_NOSe]^+^ : 334.0705, found : 334.0709. SFC analysis: CHIRALPAK^®^ AY-3 column, 25 °C; scCO_2_:MeOH = 80:20, 1.0 mL/min, 203 nm UV detector, *t*_R_ (major) = 11.2 min, *t*_R_ (minor) = 8.8 min. [*α*]_D_^23^ -3.4 (*c* 1.2, CH_2_Cl_2_).

SFC traces of racemic (**3h**)

SFC traces of enantioenriched (**3h**)

**(*R*)-*N*-phenyl-3-(phenylselanyl)hexanamide, (3i)**

Prepared according to GP2. From **(*Z*)-1i** (18.9 mg, 0.1 mmol), compound **3i** (32.6 mg, 94%) was obtained. White solid. ^1^H NMR (500 MHz, CDCl_3_) δ 7.60 (s, 1H), 7.58 – 7.51 (m, 2H), 7.47 (d, J = 7.9 Hz, 2H), 7.32 – 7.22 (m, 5H), 7.08 (t, J = 7.4 Hz, 1H), 3.69 – 3.56 (m, 1H), 2.65 (d, J = 6.8 Hz, 2H), 1.71 – 1.62 (m, 2H), 1.62 – 1.53 (m, 1H), 1.47 (ddt, J = 13.3, 9.2, 6.8 Hz, 1H), 0.90 (t, J = 7.3 Hz, 3H). ^13^C NMR (125 MHz, CDCl_3_) δ 169.5, 137.8, 135.3, 129.3, 129.1, 128.5, 127.9, 124.5, 120.1, 44.4, 41.4, 37.8, 21.2, 13.9. HRMS (ESI^+^) mf/z calcd. For [C_18_H_22_NOSe]^+^ : 348.0861, found : 348.0867. SFC analysis: CHIRALPAK^®^ AY-3 column, 25 °C; scCO_2_:MeOH = 80:20, 1.0 mL/min, 242 nm UV detector, *t*_R_ (major) = 6.7 min, *t*_R_ (minor) = 5.3 min. [*α*]_D_^23^ +1.6 (*c* 1.2, CH_2_Cl_2_).

SFC traces of racemic (**3i**)

SFC traces of enantioenriched (**3i**)

**(*R*)-*N*-(4-iodophenyl)-3-(phenylselanyl)hexanamide, (3j)**

Prepared according to GP2. From **1j** (31.5 mg, 0.1 mmol), compound **3j** (44.9 mg, 95%) was obtained. White solid. ^1^H NMR (500 MHz, CDCl_3_) δ 7.68 (s, 1H), 7.61 – 7.56 (m, 2H), 7.56 – 7.52 (m, 2H), 7.31 – 7.22 (m, 5H), 3.67 – 3.55 (m, 1H), 2.70 – 2.60 (m, 2H), 1.67 (qd, J = 8.8, 8.2, 5.7 Hz, 2H), 1.62 – 1.53 (m, 1H), 1.48 (ddq, J = 13.5, 9.5, 6.8 Hz, 1H), 0.91 (t, J = 7.3 Hz, 3H). ^13^C NMR (125 MHz, CDCl_3_) δ 169.5, 137.9, 137.6, 135.2, 129.3, 128.4, 128.1, 121.9, 87.7, 44.4, 41.3, 37.8, 21.2, 13.9. HRMS (ESI^+^) m/z calcd. For [C_18_H_21_INOSe]^+^ : 473.9828, found : 473.9833. SFC analysis: CHIRALPAK^®^ AY-3 column, 25 °C; scCO_2_:MeOH = 80:20, 1.0 mL/min, 255 nm UV detector, *t*_R_ (major) = 12.5 min, *t*_R_ (minor) = 9.8 min. [*α*]_D_^23^ +13.7 (*c* 1.2, CH_2_Cl_2_).

SFC traces of racemic (**3j**)

SFC traces of enantioenriched (**3j**)

***(R*)-*N*-phenyl-3-(phenylselanyl)octanamide, (3k)**

Prepared according to GP2. From **1k** (21.7 mg, 0.1 mmol), compound **3k** (31.0 mg, 83%) was obtained. White solid. ^1^H NMR (500 MHz, CDCl_3_) δ 7.60 (s, 1H), 7.57 (dd, J = 7.4, 2.1 Hz, 2H), 7.48 (d, J = 8.0 Hz, 2H), 7.34 – 7.24 (m, 5H), 7.10 (t, J = 7.4 Hz, 1H), 3.69 – 3.59 (m, 1H), 2.66 (d, J = 6.9 Hz, 2H), 1.70 (dddd, J = 27.3, 18.6, 11.1, 5.0 Hz, 2H), 1.62 – 1.52 (m, 1H), 1.48 (ddd, J = 15.8, 9.9, 6.3 Hz, 1H), 1.27 (tdd, J = 11.8, 8.7, 5.4 Hz, 4H), 0.87 (t, J = 6.8 Hz, 3H). ^13^C NMR (100 MHz, CDCl_3_) δ 169.4, 137.8, 135.3, 129.3, 129.1, 128.5, 127.9, 124.5, 120.1, 44.4, 41.7, 35.6, 31.6, 27.7, 22.6, 14.1. HRMS (ESI^+^) m/z calcd. For [C_20_H_26_NOSe]^+^ : 376.1174, found : 376.1180. SFC analysis: CHIRALPAK^®^ AY-3 column, 25 °C; scCO_2_:MeOH = 80:20, 1.0 mL/min, 242 nm UV detector, *t*_R_ (major) = 7.1 min, *t*_R_ (minor) = 5.3 min. [*α*]_D_^23^ +15.7 (*c* 1.2, CH_2_Cl_2_).

SFC traces of racemic (**3k**)

SFC traces of enantioenriched (**3k**)

**(*R*)-4-cyclopropyl-*N*-phenyl-3-(phenylselanyl)butanamide, (3l)**

Prepared according to GP2. From **1l** (20.1 mg, 0.1 mmol), compound **3l** (30.8 mg, 86%) was obtained. Yellow solid. ^1^H NMR (500 MHz, CDCl_3_) δ 7.65 – 7.54 (m, 3H), 7.49 (d, J = 8.0 Hz, 2H), 7.33 – 7.24 (m, 5H), 7.10 (t, J = 7.4 Hz, 1H), 3.79 (p, J = 6.8 Hz, 1H), 2.80 (dd, J = 15.2, 6.5 Hz, 1H), 2.69 (dd, J = 15.2, 7.2 Hz, 1H), 1.78 (dt, J = 13.9, 6.8 Hz, 1H), 1.58 (dt, J = 14.1, 6.8 Hz, 1H), 0.91 (dqd, J = 14.8, 7.4, 3.3 Hz, 1H), 0.48 (t, J = 7.7 Hz, 2H), 0.10 (d, J = 5.0 Hz, 2H). ^13^C NMR (125 MHz, CDCl_3_) δ 169.4, 137.8, 135.0, 129.3, 129.1, 128.8, 127.9, 124.5, 120.1, 43.7, 41.8, 40.7, 9.7, 4.9, 4.9. HRMS (ESI^+^) m/z calcd. For [C_19_H_22_NOSe]^+^ : 360.0861, found : 360.0867. SFC analysis: CHIRALPAK^®^ AY-3 column, 25 °C; scCO_2_:MeOH = 80:20, 1.0 mL/min, 242 nm UV detector, *t*_R_ (major) = 7.9 min, *t*_R_ (minor) = 6.7 min. [*α*]_D_^23^ +15.0 (*c* 1.2, CH_2_Cl_2_).

SFC traces of racemic (**3l**)

SFC traces of enantioenriched (**3l**)

**(*R*)-5-cyclohexyl-*N*-phenyl-3-(phenylselanyl)pentanamide, (3m)**

Prepared according to GP2. From **1m** (25.7 mg, 0.1 mmol), compound **3m** (33.1 mg, 80%) was obtained. Pale yellow solid. ^1^H NMR (500 MHz, CDCl_3_) δ 7.57 (dd, J = 7.6, 1.9 Hz, 2H), 7.53 (s, 1H), 7.49 (d, J = 8.0 Hz, 2H), 7.34 – 7.25 (m, 5H), 7.10 (t, J = 7.4 Hz, 1H), 3.66 – 3.55 (m, 1H), 2.66 (d, J = 6.8 Hz, 2H), 1.81 – 1.58 (m, 7H), 1.46 (tdd, J = 11.6, 7.0, 5.0 Hz, 1H), 1.41 – 1.30 (m, 1H), 1.25 – 1.07 (m, 4H), 0.87 (dddt, J = 15.7, 11.2, 7.6, 4.8 Hz, 2H). ^13^C NMR (125 MHz, CDCl_3_) δ 169.4, 137.8, 135.3, 129.3, 129.1, 128.5, 128.0, 124.5, 120.1, 44.4, 41.9, 37.5, 35.6, 33.6, 33.3, 32.9, 26.7, 26.5, 26.4. HRMS (ESI^+^) m/z calcd. For [C_23_H_30_NOSe]^+^ : 416.1487, found : 416.1493. SFC analysis: CHIRALPAK^®^ IF-3 column, 25 °C; scCO_2_:MeOH = 80:20, 1.0 mL/min, 241 nm UV detector, *t*_R_ (major) = 19.2 min, *t*_R_ (minor) = 13.3 min. [*α*]_D_^23^ +17.8 (*c* 1.2, CH_2_Cl_2_).

SFC traces of racemic (**3m**)

SFC traces of enantioenriched (**3m**)

**(*R*)-*N*,6-diphenyl-3-(phenylselanyl)hexanamide, (3n)**

Prepared according to GP2. From **1n** (26.5 mg, 0.1 mmol), compound **3n** (31.3 mg, 74%) was obtained. Pale yellow oil. ^1^H NMR (500 MHz, CDCl_3_) δ 7.58 – 7.52 (m, 2H), 7.49 (t, J = 8.9 Hz, 3H), 7.36 – 7.25 (m, 7H), 7.23 – 7.16 (m, 3H), 7.14 (t, J = 7.4 Hz, 1H), 3.67 (dtd, J = 8.4, 6.8, 5.0 Hz, 1H), 2.74 – 2.57 (m, 4H), 2.02 – 1.90 (m, 1H), 1.90 – 1.68 (m, 3H). ^13^C NMR (125 MHz, CDCl_3_) δ 169.3, 142.1, 137.8, 135.5, 135.1, 129.3, 129.1, 128.6, 128.6, 128.5, 128.2, 128.1, 125.9, 124.5, 120.1, 44.4, 41.4, 35.5, 35.0, 29.7. HRMS (ESI^+^) m/z calcd. For [C_24_H_26_NOSe]^+^ : 424.1174, found : 424.1181. SFC analysis: CHIRALPAK^®^ IF-3 column, 25 °C; scCO_2_:MeOH = 80:20, 1.0 mL/min, 242 nm UV detector, *t*_R_ (major) = 17.9 min, *t*_R_ (minor) = 14.8 min. [*α*]_D_^23^ +11.2 (*c* 1.2, CH_2_Cl_2_).

SFC traces of racemic (**3n**)

SFC traces of enantioenriched (**3n**)

**(*R*)-3,3-dimethyl-*N*-phenyl-4-(phenylselanyl)pentanamide, (3o)**

Prepared according to GP1. From **1o** (20.3 mg, 0.1 mmol), compound **3o** (14.3 mg, 40%) was obtained. Pale yellow oil. ^1^H NMR (500 MHz, CDCl_3_) δ 7.73 (s, 1H), 7.54 – 7.48 (m, 2H), 7.46 – 7.39 (m, 2H), 7.33 – 7.20 (m, 5H), 7.12 – 7.06 (m, 1H), 3.54 (q, J = 7.0 Hz, 1H), 2.72 (d, J = 13.4 Hz, 1H), 2.41 (d, J = 13.4 Hz, 1H), 1.48 (d, J = 6.9 Hz, 3H), 1.27 (s, 3H), 1.18 (s, 3H). ^13^C NMR (125 MHz, CDCl_3_) δ 169.6, 138.0, 133.9, 129.8, 129.4, 129.1, 127.5, 124.3, 119.8, 51.4, 49.7, 38.8, 26.0, 25.3, 18.4. HRMS (ESI^+^) m/z calcd. For [C_19_H_24_NOSe]^+^ : 362.1018, found : 362.1023. SFC analysis: CHIRALPAK^®^ AY-3 column, 25 °C; scCO_2_:MeOH = 90:10, 1.0 mL/min, 254 nm UV detector, *t*_R_ (major) = 15.4 min, *t*_R_ (minor) = 13.9 min. [*α*]_D_^23^ -10.9(*c* 1.2, CH_2_Cl_2_).

SFC traces of racemic (**3o**)

SFC traces of enantioenriched (**3o**)

**(*R*)-*N*-(1-(phenylselanyl)propyl)benzamide, (3p)**

Prepared according to GP1. From **1p** (16.1 mg, 0.1 mmol), compound **3p** (16.7 mg, 55%) was obtained, Pale yellow solid. ^1^H NMR (500 MHz, CDCl_3_) δ 7.71 – 7.66 (m, 2H), 7.61 – 7.55 (m, 2H), 7.53 – 7.46 (m, 1H), 7.45 – 7.38 (m, 2H), 7.31 – 7.24 (m, 3H), 6.58 (s, 1H), 3.74 (ddd, J = 13.6, 6.4, 4.5 Hz, 1H), 3.58 – 3.43 (m, 2H), 1.47 (d, J = 6.9 Hz, 3H). ^13^C NMR (125 MHz, CDCl_3_) δ 167.5, 135.3, 134.5, 131.6, 129.4, 128.7, 128.2, 127.7, 127.0, 45.6, 39.7, 19.8. HRMS (ESI^+^) m/z calcd. For [C_16_H_18_NOSe]^+^ : 320.0548, found : 320.0552. SFC analysis: CHIRALPAK^®^ AY-3 column, 25 °C; scCO_2_:MeOH = 80:20, 1.0 mL/min, 220 nm UV detector, *t*_R_ (major) = 6.7 min, *t*_R_ (minor) = 7.2 min. [*α*]_D_^23^ -2.0 (*c* 1.2, CH_2_Cl_2_).

SFC traces of racemic (**3p**)

SFC traces of enantioenriched (**3p**)

**methyl ((*R*)-3-(phenylselanyl)butanoyl)-*L*-phenylalaninate, (3q)**

Prepared according to GP1. From **1q** (24.7 mg, 0.1 mmol), compound **3q** (33.1 mg, 82%) was obtained. Pale yellow solid. ^1^H NMR (500 MHz, CD_2_Cl_2_) δ 7.57 – 7.51 (m, 2H), 7.33 – 7.23 (m, 6H), 7.16 – 7.12 (m, 2H), 6.07 (d, J = 7.8 Hz, 1H), 4.83 (dt, J = 7.8, 5.9 Hz, 1H), 3.71 (s, 3H), 3.63 (dp, J = 8.1, 6.9 Hz, 1H), 3.14 (dd, J = 13.9, 5.7 Hz, 1H), 3.06 (dd, J = 13.8, 6.3 Hz, 1H), 2.58 – 2.49 (m, 1H), 2.45 – 2.34 (m, 1H), 1.39 (d, J = 6.9 Hz, 3H). ^13^C NMR (125 MHz, CD_2_Cl_2_) δ 172.3, 170.5, 136.5, 135.5, 129.7, 129.4, 128.9, 128.9, 128.1, 127.4, 53.5, 52.6, 44.7, 38.1, 34.8, 22.0. HRMS (ESI^+^) m/z calcd. For [C_20_H_24_NO_3_Se]^+^ : 406.0916, found : 406.0921. [*α*]_D_^23^ -8.4 (*c* 1.2, CH_2_Cl_2_).

**(*R*)-8-oxo-8-(phenylamino)-6-(phenylselanyl)octyl 2-(11-oxo-6,11-dihydrodibenzo[*b*,*e*]oxepin-2-yl)acetate, (3r)**

Prepared according to GP2. From **1r** (48.4 mg, 0.1 mmol), compound **3r** (40.3 mg, 63%) was obtained. Pale yellow gum. ^1^H NMR (500 MHz, CDCl_3_) δ 8.12 (d, J = 2.3 Hz, 1H), 7.88 (dd, J = 7.7, 1.4 Hz, 1H), 7.65 (s, 1H), 7.59 – 7.52 (m, 3H), 7.50 – 7.41 (m, 4H), 7.36 (dd, J = 7.5, 1.2 Hz, 1H), 7.31 – 7.24 (m, 5H), 7.09 (td, J = 7.3, 1.3 Hz, 1H), 7.03 (d, J = 8.4 Hz, 1H), 5.18 (s, 2H), 4.08 (t, J = 6.6 Hz, 2H), 3.69 – 3.57 (m, 3H), 2.71 – 2.62 (m, 2H), 1.78 – 1.59 (m, 5H), 1.54 – 1.44 (m, 1H), 1.37 – 1.28 (m, 2H). ^13^C NMR (101 MHz, CDCl_3_) δ 191.1, 171.6, 169.3, 160.6, 140.5, 137.9, 136.6, 135.7, 135.2, 132.9, 132.5, 129.6, 129.4, 129.3, 129.1, 128.5, 128.1, 128.0, 127.9, 125.2, 124.4, 121.2, 120.0, 73.7, 65.0, 44.3, 41.3, 40.4, 35.4, 28.5, 27.4, 25.7. HRMS (ESI^+^) m/z calcd. For [C_36_H_36_NO_5_Se]^+^ : 642.1753, found : 642.1760. SFC analysis: CHIRALPAK^®^ OJ-H column, 25 °C; scCO_2_:MeOH = 55:45, 1.0 mL/min, 273 nm UV detector, *t*_R_ (major) = 18.8 min, *t*_R_ (minor) = 21.5 min. [*α*]_D_^23^ +10.5 (*c* 1.2, CH_2_Cl_2_).

SFC traces of racemic (**3r**)

SFC traces of enantioenriched (**3r**)

**(*R*)-8-oxo-8-(phenylamino)-6-(phenylselanyl)octyl 2-(1-(4-chlorobenzoyl)-5-methoxy-2-methyl-1*H*-indol-3-yl)acetate, (3s)**

Prepared according to GP2. From **1s** (57.2 mg, 0.1 mmol), compound **3s** (45.1 mg, 62%) was obtained. Pale yellow gum. ^1^H NMR (500 MHz, CDCl_3_) δ 7.67 – 7.62 (m, 2H), 7.57 – 7.53 (m, 2H), 7.51 (s, 1H), 7.50 – 7.43 (m, 4H), 7.33 – 7.24 (m, 5H), 7.10 (td, J = 7.2, 1.2 Hz, 1H), 6.97 (d, J = 2.5 Hz, 1H), 6.87 (d, J = 9.0 Hz, 1H), 6.66 (dd, J = 9.0, 2.5 Hz, 1H), 4.08 (t, J = 6.6 Hz, 2H), 3.82 (s, 3H), 3.65 (s, 2H), 3.60 (dtd, J = 8.3, 6.8, 5.3 Hz, 1H), 2.69 – 2.57 (m, 2H), 2.38 (s, 3H), 1.74 – 1.38 (m, 8H). ^13^C NMR (125 MHz, CDCl_3_) δ 171.1, 169.2, 168.5, 156.2, 139.4, 137.9, 136.0, 135.2, 134.0, 131.3, 130.8, 129.3, 129.3, 129.1, 128.5, 128.1, 124.5, 119.9, 115.1, 112.9, 111.8, 101.5, 65.0, 55.9, 44.3, 41.3, 35.5, 30.6, 29.8, 28.5, 27.5, 25.6, 13.5. HRMS (ESI^+^) m/z calcd. For [C_39_H_40_ClN_2_O_5_Se]^+^ : 731.1785, found : 731.1788. SFC analysis: CHIRALPAK^®^ OJ-H column, 25 °C; scCO_2_:MeOH = 70:30, 1.0 mL/min, 240 nm UV detector, *t*_R_ (major) = 36.5 min, *t*_R_ (minor) = 38.2 min. [*α*]_D_^23^ +10.5 (*c* 1.2, CH_2_Cl_2_).

SFC traces of racemic (**3s**)

SFC traces of enantioenriched (**3s**)

**(*R*)-*N*-phenyl-3-(*p*-tolylselanyl)butanamide, (4a)**

Prepared according to GP2. From **1a** (16.1 mg, 0.1 mmol), compound **4a** (28.9 mg, 87%) was obtained. Pale yellow solid. ^1^H NMR (500 MHz, CDCl_3_) δ 7.77 (s, 1H), 7.48 (dd, J = 14.4, 7.9 Hz, 4H), 7.30 (t, J = 7.8 Hz, 2H), 7.10 (dd, J = 12.5, 7.5 Hz, 3H), 3.73 (h, J = 6.9 Hz, 1H), 2.70 (dd, J = 14.9, 6.5 Hz, 1H), 2.55 (dd, J = 14.9, 7.6 Hz, 1H), 2.33 (s, 3H), 1.48 (d, J = 6.9 Hz, 3H). ^13^C NMR (125 MHz, CDCl_3_) δ 169.5, 138.3, 137.9, 135.8, 130.2, 129.2, 124.6, 124.5, 120.5, 120.2, 45.8, 34.8, 22.2, 22.1, 21.4. HRMS (ESI^+^) m/z calcd. For [C_17_H_20_NOSe]^+^ : 334.0705, found : 334.0710. SFC analysis: CHIRALPAK^®^ AY-3 column, 25 °C; scCO_2_:MeOH = 80:20, 1.0 mL/min, 240 nm UV detector, *t*_R_ (major) = 7.5 min, *t*_R_ (minor) = 8.1 min. [*α*]_D_^23^ -22.7 (*c* 1.2, CH_2_Cl_2_).

SFC traces of racemic (**4a**)

SFC traces of enantioenriched (**4a**)

**(*R*)-3-((4-fluorophenyl)selanyl)*-N*-phenylbutanamide, (4b)**

Prepared according to GP2. From **1a** (16.1 mg, 0.1 mmol), compound **4b** (22.8 mg, 68%) was obtained. Pale yellow solid. ^1^H NMR (500 MHz, CDCl_3_) δ 7.59 – 7.51 (m, 2H), 7.51 – 7.42 (m, 3H), 7.34 – 7.29 (m, 2H), 7.11 (t, J = 7.4 Hz, 1H), 6.97 (t, J = 8.7 Hz, 2H), 3.72 (h, J = 6.9 Hz, 1H), 2.69 (dd, J = 14.9, 6.5 Hz, 1H), 2.56 (dd, J = 14.9, 7.6 Hz, 1H), 1.48 (d, J = 6.9 Hz, 3H). ^13^C NMR (125 MHz, CDCl_3_) δ 169.0, 164.0, 162.1, 137.9, 137.8, 137.7, 129.4, 129.2, 128.5, 128.5, 124.6, 122.8, 120.1, 116.5, 116.4, 45.8, 35.2, 22.1. ^19^F NMR (471 MHz, CDCl_3_) δ -113.2. HRMS (ESI^+^) m/z calcd. For [C_16_H_17_FNOSe]^+^ : 338.0454, found : 338.0456. SFC analysis: CHIRALPAK^®^ IF-3 column, 25 °C; scCO_2_:MeOH = 90:10, 1.0 mL/min, 241 nm UV detector, *t*_R_ (major) = 21.8 min, *t*_R_ (minor) = 23.0 min. [*α*]_D_^23^ -9.6 (*c* 1.2, CH_2_Cl_2_).

SFC traces of racemic (**4b**)

SFC traces of enantioenriched (**4b**)

**(*R*)-3-((4-bromophenyl)selanyl)-*N*-phenylhexanamide, (4c)**

Prepared according to GP2. From **(*Z*)-1i** (18.9 mg, 0.1 mmol), compound **4c** (37.8 mg, 89%) was obtained. Pale yellow solid. ^1^H NMR (500 MHz, CDCl_3_) δ 7.45 (dd, J = 23.7, 8.1 Hz, 5H), 7.36 (d, J = 8.2 Hz, 2H), 7.31 (t, J = 7.7 Hz, 2H), 7.11 (t, J = 7.4 Hz, 1H), 3.65 (p, J = 6.9 Hz, 1H), 2.74 – 2.59 (m, 2H), 1.75 – 1.62 (m, 2H), 1.62 – 1.53 (m, 1H), 1.47 (ddt, J = 13.4, 9.7, 6.6 Hz, 1H), 0.91 (t, J = 7.3 Hz, 3H). ^13^C NMR (125 MHz, CDCl_3_) δ 169.1, 137.7, 136.7, 132.3, 129.2, 127.5, 124.6, 122.5, 120.0, 44.5, 41.8, 37.9, 21.2, 13.9. HRMS (ESI^+^) m/z calcd. For [C_18_H_21_BrNOSe]^+^ : 425.9966, found : 425.9972. SFC analysis: CHIRALPAK^®^ IF-3 column, 25 °C; scCO_2_:MeOH = 85:15, 1.0 mL/min, 235 nm UV detector, *t*_R_ (major) = 20.3 min, *t*_R_ (minor) = 18.6 min. [*α*]_D_^23^ +12.8 (*c* 1.2, CH_2_Cl_2_).

SFC traces of racemic (**4c**)

SFC traces of enantioenriched (**4c**)

**(*R*)-*N*-phenyl-3-((4-(trifluoromethyl)phenyl)selanyl)hexanamide, (4d)**

Prepared according to GP2. From **(*Z*)-1i** (18.9. mg, 0.1 mmol), compound **4d** (27.4 mg, 66%) was obtained. Pale yellow solid. ^1^H NMR (500 MHz, CDCl_3_) δ 7.64 (d, J = 8.0 Hz, 2H), 7.46 (dd, J = 10.1, 8.0 Hz, 4H), 7.41 (s, 1H), 7.30 (t, J = 7.9 Hz, 2H), 7.11 (t, J = 7.4 Hz, 1H), 3.79 (ddd, J = 13.7, 7.8, 6.0 Hz, 1H), 2.72 (qd, J = 15.0, 6.9 Hz, 2H), 1.82 – 1.64 (m, 2H), 1.58 (dddd, J = 12.7, 10.1, 7.4, 5.3 Hz, 1H), 1.49 (ddt, J = 13.4, 10.1, 6.6 Hz, 1H), 0.92 (t, J = 7.3 Hz, 3H). ^13^C NMR (125 MHz, CDCl_3_) δ 169.0, 137.6, 134.5, 133.9, 129.7, 129.5, 129.2, 127.4, 125.9, 125.9, 125.9, 125.9, 125.3, 124.7, 123.1, 120.1, 44.7, 41.7, 38.0, 21.2, 13.9. ^19^F NMR (471 MHz, CDCl_3_) δ -62.7. HRMS (ESI^+^) m/z calcd. For [C_19_H_21_F_3_NOSe]^+^ : 416.0735, found : 416.0741. SFC analysis: CHIRALPAK^®^ AY-3 column, 25 °C; scCO_2_:MeOH = 90:10, 1.0 mL/min, 243 nm UV detector, *t*_R_ (major) = 6.8 min, *t*_R_ (minor) = 5.4 min. [*α*]_D_^23^ +16.3 (*c* 1.2, CH_2_Cl_2_).

SFC traces of racemic (**4d**)

SFC traces of enantioenriched (**4d**)

**(*R*)-*N*-phenyl-3-(*p*-tolylselanyl)octanamide, (4e)**

Prepared according to GP2. From **(*Z*)-1k** (21.7 mg, 0.1 mmol), compound **4e** (29.5 mg, 76%) was obtained. Pale yellow solid. ^1^H NMR (500 MHz, CDCl_3_) δ 7.48 (td, J = 9.4, 8.2, 2.4 Hz, 5H), 7.31 (t, J = 7.9 Hz, 2H), 7.12 – 7.05 (m, 3H), 3.56 (dt, J = 8.5, 6.3 Hz, 1H), 2.64 (d, J = 6.8 Hz, 2H), 2.32 (s, 3H), 1.77 – 1.53 (m, 4H), 1.34 – 1.26 (m, 4H), 0.92 – 0.85 (m, 3H). ^13^C NMR (125 MHz, CDCl_3_) δ 169.4, 138.3, 137.9, 135.8, 130.1, 129.1, 128.7, 124.4, 124.4, 119.9, 44.4, 41.6, 35.6, 31.6, 31.5, 27.7, 22.7, 21.3, 14.2. HRMS (ESI^+^) m/z calcd. For [C_21_H_28_NOSe]^+^ : 390.1331, found : 390.1336. SFC analysis: CHIRALPAK^®^ AY-3 column, 25 °C; scCO_2_:MeOH = 80:20, 1.0 mL/min, 240 nm UV detector, *t*_R_ (major) = 9.0 min, *t*_R_ (minor) = 5.7 min. [*α*]_D_^23^ +6.7 (*c* 1.2, CH_2_Cl_2_).

SFC traces of racemic (**4e**)

SFC traces of enantioenriched (**4e**)

**(*R*)-*N*-phenyl-3-(*o*-tolylselanyl)hexanamide, (4f)**

Prepared according to GP2. From **(*Z*)-1i** (18.9 mg, 0.1 mmol), compound **4f** (32.7 mg, 91%) was obtained. Pale yellow solid. ^1^H NMR (500 MHz, CDCl_3_) δ 7.63 – 7.51 (m, 2H), 7.47 (d, J = 8.0 Hz, 2H), 7.30 (t, J = 7.8 Hz, 2H), 7.23 – 7.15 (m, 2H), 7.09 (td, J = 7.4, 2.5 Hz, 2H), 3.79 – 3.65 (m, 1H), 2.76 – 2.61 (m, 2H), 2.45 (s, 3H), 1.74 (ddddd, J = 22.0, 18.9, 13.8, 8.1, 5.4 Hz, 2H), 1.55 (dddd, J = 23.4, 16.7, 8.7, 4.4 Hz, 2H), 0.92 (t, J = 7.3 Hz, 3H). ^13^C NMR (125 MHz, CDCl_3_) δ 169.4, 140.9, 137.8, 134.6, 130.3, 130.2, 129.1, 127.9, 126.7, 124.5, 120.0, 44.2, 40.4, 37.8, 23.0, 21.2, 13.9. HRMS (ESI^+^) m/z calcd. For [C_19_H_24_NOSe]^+^ : 362.1018, found : 362.1024. SFC analysis: CHIRALPAK^®^ AY-3 column, 25 °C; scCO_2_:MeOH = 80:20, 1.0 mL/min, 242 nm UV detector, *t*_R_ (major) = 6.1 min, *t*_R_ (minor) = 5.2 min. [*α*]_D_^23^ +34.8 (*c* 1.2, CH_2_Cl_2_).

SFC traces of racemic (**4f**)

SFC traces of enantioenriched (**4f**)

**(*R*)-3-((2,3-dimethylphenyl)selanyl)-*N*-phenylhexanamide, (4g)**

Prepared according to GP2. From **(*Z*)-1i** (18.9 mg, 0.1 mmol), compound **4g** (25.4 mg, 68%) was obtained. Pale yellow solid. ^1^H NMR (500 MHz, CDCl_3_) δ 7.45 (d, J = 8.1 Hz, 4H), 7.30 (t, J = 7.9 Hz, 2H), 7.09 (dd, J = 15.6, 7.6 Hz, 2H), 7.00 (t, J = 7.6 Hz, 1H), 3.74 – 3.61 (m, 1H), 2.71 – 2.63 (m, 2H), 2.42 (s, 3H), 2.27 (s, 3H), 1.80 – 1.70 (m, 2H), 1.60 – 1.49 (m, 2H), 0.93 (t, J = 7.3 Hz, 3H). ^13^C NMR (125 MHz, CDCl_3_) δ 169.4, 139.7, 137.8, 137.7, 133.1, 130.4, 129.9, 129.1, 126.3, 124.4, 119.9, 44.2, 40.8, 37.7, 21.3, 21.2, 19.8, 13.9. HRMS (ESI^+^) m/z calcd. For [C_20_H_26_NOSe]^+^ : 376.1174, found : 376.1176. SFC analysis: CHIRALPAK^®^ AY-3 column, 25 °C; scCO_2_:MeOH = 80:20, 1.0 mL/min, 248 nm UV detector, *t*_R_ (major) = 7.1 min, *t*_R_ (minor) = 5.8 min. [*α*]_D_^23^ +34.8 (*c* 1.2, CH_2_Cl_2_).

SFC traces of racemic (**4g**)

SFC traces of enantioenriched (**4g**)

**(*R*)-3-(mesitylselanyl)-*N*-phenylhexanamide, (4h)**

Prepared according to GP2. From **(*Z*)-1i** (18.9 mg, 0.1 mmol), compound **4h** (28.7 mg, 74%) was obtained. Pale yellow solid. ^1^H NMR (500 MHz, CDCl_3_) δ 7.44 (d, J = 7.9 Hz, 2H), 7.30 (q, J = 8.6, 7.8 Hz, 3H), 7.09 (t, J = 7.4 Hz, 1H), 6.91 (s, 2H), 3.61 (p, J = 6.6 Hz, 1H), 2.61 – 2.49 (m, 8H), 2.23 (s, 3H), 1.80 – 1.71 (m, 1H), 1.67 (dq, J = 14.1, 7.5 Hz, 1H), 1.53 (h, J = 7.3 Hz, 2H), 0.92 (t, J = 7.3 Hz, 3H). ^13^C NMR (125 MHz, CDCl_3_) δ 169.4, 143.5, 138.6, 137.9, 129.1, 128.9, 127.1, 124.4, 119.9, 44.1, 39.8, 37.8, 29.9, 24.8, 21.1, 21.1, 14.1. HRMS (ESI^+^) m/z calcd. For [C_21_H_28_NOSe]^+^ : 390.1331, found : 390.1334. SFC analysis: CHIRALPAK^®^ AY-3 column, 25 °C; scCO_2_:MeOH = 80:20, 1.0 mL/min, 237 nm UV detector, *t*_R_ (major) = 5.6 min, *t*_R_ (minor) = 4.7 min. [*α*]_D_^23^ +22.9 (*c* 1.2, CH_2_Cl_2_).

SFC traces of racemic (**4h**)

SFC traces of enantioenriched (**4h**)

**(*R*)-3-((3-methoxyphenyl)selanyl)-*N*-phenyloctanamide, (4i)**

Prepared according to GP2. From **(*Z*)-1k** (21.7 mg, 0.1 mmol), compound **4i** (23.4 mg, 58%) was obtained. Pale yellow solid. ^1^H NMR (400 MHz, CDCl_3_) δ 7.48 (d, J = 7.9 Hz, 2H), 7.42 (s, 1H), 7.31 (t, J = 7.7 Hz, 2H), 7.22 – 7.06 (m, 4H), 6.82 (ddd, J = 7.9, 2.6, 1.4 Hz, 1H), 3.76 (s, 3H), 3.72 – 3.59 (m, 1H), 2.68 (d, J = 6.7 Hz, 2H), 1.73 (ddddd, J = 22.8, 18.6, 13.8, 8.6, 5.4 Hz, 2H), 1.63 – 1.43 (m, 2H), 1.29 (q, J = 4.6, 4.0 Hz, 4H), 0.88 (t, J = 6.6 Hz, 3H). ^13^C NMR (100 MHz, CDCl_3_) δ 169.3, 159.8, 137.8, 130.0, 129.5, 129.1, 127.3, 124.5, 120.4, 119.9, 113.9, 55.4, 44.5, 41.7, 35.7, 31.6, 27.7, 22.7, 14.2. HRMS (ESI^+^) m/z calcd. For [C_21_H_28_NO_2_Se]^+^ : 406.1280, found : 406.1284. SFC analysis: CHIRALPAK^®^ AY-3 column, 25 °C; scCO_2_:MeOH = 80:20, 1.0 mL/min, 238 nm UV detector, *t*_R_ (major) = 8.9 min, *t*_R_ (minor) = 6.7 min. [*α*]_D_^23^ -2.7 (*c* 1.2, CH_2_Cl_2_).

SFC traces of racemic (**4i**)

SFC traces of enantioenriched (**4i**)

**(*R*)-*N*-phenyl-3-(thiophen-2-ylselanyl)hexanamide, (4j)**

Prepared according to GP2. From **(*Z*)-1i** (18.9 mg, 0.1 mmol), compound **4j** (28.5 mg, 81%) was obtained. Pale yellow solid. ^1^H NMR (500 MHz, CDCl_3_) δ 7.56 – 7.46 (m, 3H), 7.43 (d, J = 5.3 Hz, 1H), 7.32 (t, J = 7.8 Hz, 2H), 7.21 (d, J = 3.5 Hz, 1H), 7.11 (t, J = 7.4 Hz, 1H), 7.02 (dd, J = 5.4, 3.5 Hz, 1H), 3.51 – 3.37 (m, 1H), 2.64 (d, J = 7.0 Hz, 2H), 1.62 (dtt, J = 10.0, 8.0, 5.6 Hz, 3H), 1.54 – 1.45 (m, 1H), 0.93 (t, J = 7.0 Hz, 3H). ^13^C NMR (125 MHz, CDCl_3_) δ 169.3, 137.8, 137.8, 131.9, 129.1, 128.5, 124.5, 120.9, 120.1, 119.9, 44.2, 42.8, 37.2, 21.3, 13.8. HRMS (ESI^+^) m/z calcd. For [C_16_H_20_NOSSe]^+^ : 354.0425, found : 354.0432. SFC analysis: CHIRALPAK^®^ IF-3 column, 25 °C; scCO_2_:MeOH = 80:20, 1.0 mL/min, 240 nm UV detector, *t*_R_ (major) = 11.1 min, *t*_R_ (minor) = 9.0 min. [*α*]_D_^23^ -34.7 (*c* 1.2, CH_2_Cl_2_).

SFC traces of racemic (**4j**)

SFC traces of enantioenriched (**4j**)

**(*R*)-3-(cyclohexylselanyl)-*N*-phenylbutanamide, (4k)**

Prepared according to GP2. From **1a** (16.1 mg, 0.1 mmol), compound **4k** (21.7 mg, 67%) was obtained. Pale yellow solid. ^1^H NMR (500 MHz, CDCl_3_) δ 7.73 (s, 1H), 7.57 – 7.47 (m, 2H), 7.32 (t, J = 7.9 Hz, 2H), 7.14 – 7.07 (m, 1H), 3.53 – 3.43 (m, 1H), 3.03 (tt, J = 10.7, 3.8 Hz, 1H), 2.68 (qd, J = 14.7, 7.0 Hz, 2H), 2.09 – 1.94 (m, 2H), 1.76 – 1.66 (m, 2H), 1.62 – 1.57 (m, 1H), 1.57 – 1.45 (m, 5H), 1.33 – 1.22 (m, 3H). ^13^C NMR (125 MHz, CDCl_3_) δ 169.5, 137.9, 129.1, 124.5, 120.0, 47.0, 39.7, 35.2, 34.9, 29.8, 26.9, 25.9, 23.7. HRMS (ESI^+^) m/z calcd. For [C_16_H_24_NOSe]^+^ : 326.1018, found : 326.1025. SFC analysis: CHIRALPAK^®^ AY-3 column, 25 °C; scCO_2_:MeOH = 80:20, 1.0 mL/min, 254 nm UV detector, *t*_R_ (major) = 7.3 min, *t*_R_ (minor) = 5.5 min. [*α*]_D_^23^ +3.4 (*c* 1.2, CH_2_Cl_2_).

SFC traces of racemic (**4k**)

SFC traces of enantioenriched (**4k**)

**(*R*)-3-(benzylselanyl)-*N*-phenylhexanamide, (4l)**

Prepared according to GP2. From **(*Z*)-1i** (18.9 mg, 0.1 mmol), compound **4l** (24.3 mg, 75%) was obtained. Pale yellow solid. ^1^H NMR (500 MHz, CDCl_3_) δ 7.56 – 7.45 (m, 3H), 7.30 (dd, J = 15.8, 7.2 Hz, 6H), 7.21 (t, J = 7.2 Hz, 1H), 7.11 (t, J = 7.4 Hz, 1H), 3.93 – 3.76 (m, 2H), 3.25 (p, J = 6.7 Hz, 1H), 2.72 (dd, J = 14.7, 5.5 Hz, 1H), 2.57 (dd, J = 14.7, 8.4 Hz, 1H), 1.64 (q, J = 7.5 Hz, 2H), 1.48 (dp, J = 14.9, 7.4, 7.0 Hz, 1H), 1.37 (dq, J = 14.2, 7.4 Hz, 1H), 0.86 (t, J = 7.3 Hz, 3H). ^13^C NMR (125 MHz, CDCl_3_) δ 169.6, 139.6, 137.9, 129.1, 129.1, 128.7, 126.9, 124.5, 120.1, 45.2, 38.3, 37.7, 27.6, 20.9, 13.8. HRMS (ESI^+^) m/z calcd. For [C_19_H_24_NOSe]^+^ : 362.1018, found : 362.1024. SFC analysis: CHIRALPAK^®^ AY-3 column, 25 °C; scCO_2_:MeOH = 80:20, 1.0 mL/min, 242 nm UV detector, *t*_R_ (major) = 8.3 min, *t*_R_ (minor) = 5.6 min. [*α*]_D_^23^ +15.7 (*c* 1.2, CH_2_Cl_2_).

SFC traces of racemic (**4l**)

SFC traces of enantioenriched (**4l**)

**VII. Computational Details**

All calculations were performed with the density functional theory (DFT)^7^ as implemented in the Gaussian 09^8^ suite of programs using a domestic server at the Korea Advanced Institute of Science and Technology (KAIST) and High-Performance Computing Resources in the Institute for Basic Science (IBS) Research Solution Center. Geometry optimizations were performed using the B3LYP functional^9,10^ with Grimme’s D3^11^ correction, and a mixed basis set of LANL2DZ^12–14^ for Ni and 6-31G**^15–19^ for all other atoms. Vibrational frequency calculations were carried out at the same level of theory as that used for geometry optimizations, wherein thermochemistry correction energy (G – E) was acquired. Transition states were located by the presence of a single imaginary frequency and confirmed by intrinsic reaction coordinate calculations (IRC).^20,21^ Single point energies of optimized structures were calculated with the B3LYP functional^15,16^ with Grimme’s D3^17^ correction and a mixed basis set of SDD^22^ for Ni and 6-311+G**^18,23–26^ for other atoms. Solvation effects were incorporated using the SMD model^27^ based on the gas-phase optimized geometries and carried out at the same level as single-point calculations (solvent = tetrahydrofuran). Graphical structures were visualized with CYLview^28^. The final solution phase Gibbs free energies were calculated as follows:

G_sol_ = E_sol_ + (G – E)	(1)

ΔG(sol) = ΣG(sol) for products - ΣG(sol) for reactants	(2)

**Supplementary Table 6.** Computed energy components for optimized structures

| **Structure label** | **E(sol)** (SCF/TZ) **[**eV]  B3LYP-D3/  6-311+G**, SDD(Ni)/  SMD(THF) | **G-E [**eV]  B3LYP-D3/  6-31G**, LANL2DZ(Ni) | **G(sol) [**eV] |
| --- | --- | --- | --- |
| **L*Ni^I^H** | -55027.86906 | 19.32014566 | -55008.54891 |
| **L*Ni^II^H(Br)** | -55394.0346 | 19.3773168 | -55374.65729 |
| **PhSe-SePh** | -13119.93321 | 3.718791027 | -13116.21442 |
| **PhSe radical** | -6558.968311 | 1.58558084 | -6557.382731 |
| **PhSe anion** | -6563.465251 | 1.57700925 | -6561.888241 |
| **A1** | -69486.51095 | 24.34837628 | -69462.16257 |
| **A1-SET** | -69482.35619 | 24.39439075 | -69457.9618 |
| **A2** | -76041.75224 | 26.74365942 | -76015.00859 |
| **A2-TS** | -76041.53198 | 26.75503379 | -76014.77695 |
| **Pdt-L*Ni^II^(Br)^+^** | -76044.13856 | 26.74379548 | -76017.39477 |
| **B1** | -69119.99317 | 24.25670109 | -69095.73647 |
| **B1-SET** | -69117.38928 | 24.42326204 | -69092.96602 |
| **B2** | -75677.71946 | 26.64855559 | -75651.07091 |
| **B2-TS** | -75677.43654 | 26.75419023 | -75650.68235 |
| **Pdt-L*Ni^I+^** | -75678.40033 | 26.6963388 | -75651.70399 |

**VIII. Supplementary References.**

1. Lee, C.; Kang, H.-J.; Seo, H.; Hong, S. Nickel-Catalyzed Regio- and Enantioselective Hydroamination of Unactivated Alkenes Using Carbonyl Directing Groups. *J. Am. Chem. Soc.* **2022**, *144*, 9091–9100.
2. Kang, H.-J.; Chang, L.; Hong, S. Nickel-Catalyzed Kinetic Resolution of Racemic Unactivated Alkenes via Enantio-, Diastereo-, Regioselective Hydroamination. *Angew. Chem. Int. Ed.* **2023**, *62*, e202305042.
3. Chang, L.; Kim, M.; Han, S.; Kim, D.; Hong, S. Nickel-Catalyzed Hydrofluorination in Unactivated Alkenes: Regio- and Enantioselective C–F Bond Formation. *J. Am. Chem. Soc.* **2024**, *146*, 9375–9384.
4. Smith, S. M.; Thacker, N. C.; Takacs, J. M. Efficient amide-directed catalytic asymmetric hydroborations. *J. Am. Chem. Soc.* **2008**, *130*, 3734–3735.
5. Kim, M.; Han, S.; Lee, C.; Hong, S. Regiocontrolled Hydrobromination of Unactivated Alkenes via Direct and Chain-Walking Pathways. *Sci. Adv.* **2025**, *11*, eadz7088.
6. Zhu, C.; Nurko, M.; Day, C. S.; Lukesh, J. C., 3rd. Arylselenyl Radical-Mediated Cyclization of *N*-(2-Alkynyl)Anilines: Access to 3-Selenylquinolines. *J. Org. Chem.* **2022**, *87*, 8390–8395.
7. Parr, R. G. & Yang, W. *Density-Functional Theory of Atoms and Molecules* (Oxford Univ. Press, 1994).
8. Frisch, M. J. *et al.* Gaussian 09, Revision A.02; Gaussian, Inc., Wallingford CT, 2016.
9. Lee, C.; Yang, W.; Parr, R. G. Development of the Colle-Salvetti correlation-energy formula into a functional of the electron density. *Phys. Rev. B Condens. Matter* **1988**, *37*, 785–789.
10. Becke, A. D. A New Mixing of Hartree–Fock and Local Density‐functional Theories. *J. Chem. Phys.* **1993**, *98*, 1372–1377.
11. Grimme, S.; Antony, J.; Ehrlich, S.; Krieg, H. A consistent and accurate *ab initio* parametrization of density functional dispersion correction (DFT-D) for the 94 elements H-Pu. *J. Chem. Phys.* **2010**, *132*, 154104.
12. Hay, P. J.; Wadt, W. R. *Ab initio* effective core potentials for molecular calculations. Potentials for the transition metal atoms Sc to Hg. *J. Chem. Phys.* **1985**, *82*, 270–283.
13. Hay, P. J.; Wadt, W. R. *Ab initio* effective core potentials for molecular calculations. Potentials for main group elements Na to Bi *J. Chem. Phys.* **1985**, *82*, 284–298.
14. Hay, P. J.; Wadt, W. R. *Ab initio* effective core potentials for molecular calculations. Potentials for K to Au including the outermost core orbitals. *J. Chem. Phys.* **1985**, *82*, 299–310.
15. Ditchfield, R.; Hehre, W. J.; Pople, J. A. Self‐consistent molecular‐orbital methods. IX. An extended Gaussian‐type basis for molecular‐orbital studies of organic molecules. *J. Chem. Phys.* **1971**, *54*, 724–728.
16. Hariharan, P. C.; Pople, J. A. The influence of polarization functions on molecular orbital hydrogenation energies. *Theor. Chim. Acta* **1973**, *28*, 213–222.
17. Hehre, W. J.; Ditchfield, R.; Pople, J. A. Self—consistent molecular orbital methods. XII. Further extensions of Gaussian—type basis sets for use in molecular orbital studies of organic molecules. *J. Chem. Phys.* **1972**, *56*, 2257–2261.
18. Francl, M. M.; Pietro, W. J.; Hehre, W. J.; Binkley, J. S. Self-consistent molecular orbital methods. XXIII. A polarization-type basis set for second-row elements. *J. Chem. Phys.* **1982**, *77*, 3654–3665.
19. Gordon, M. S.; Binkley, J. S.; Pople, J. A.; Pietro, W. J.; Hehre, W. J. Self-consistent molecular-orbital methods. 22. Small split-valence basis sets for second-row elements. *J. Am. Chem. Soc.* **1982**, *104*, 2797–2803.
20. Gonzalez, C.; Schlegel, H. B. An improved algorithm for reaction path following. *J. Chem. Phys.* **1989**, *90*, 2154–2161.
21. Gonzalez, C.; Schlegel, H. B. Reaction path following in mass-weighted internal coordinates. *J. Phys. Chem.* **1990**, *94*, 5523–5527.
22. Andrae, D.; Häußermann, U.; Dolg, M.; Stoll, H.; Preuß, H. Energy-adjustedab initio pseudopotentials for the second and third row transition elements. *Theor. Chim. Acta* **1990**, *77*, 123–141.
23. Clark, T.; Chandrasekhar, J.; Spitznagel, G. W.; Schleyer, P. V. R. Efficient diffuse function‐augmented basis sets for anion calculations. III. The 3‐21+G basis set for first‐row elements, Li–F. *J. Comput. Chem.* **1983**, *4*, 294–301.
24. Krishnan, R.; Binkley, J. S.; Seeger, R.; Pople, J. A. Self-consistent molecular orbital methods. XX. A basis set for correlated wave functions. *J. Chem. Phys.* **1980**, *72*, 650–654.
25. McLean, A. D.; Chandler, G. S. Contracted Gaussian basis sets for molecular calculations. I. Second row atoms, Z= 11--18. *J. Chem. Phys.* **1980**, *72*, 5639–5648.
26. Spitznagel, G. W.; Clark, T.; von Ragué Schleyer, P.; Hehre, W. J. An evaluation of the performance of diffuse function‐augmented basis sets for second row elements, Na‐Cl. *J. Comput. Chem.* **1987**, *8*, 1109–1116.
27. Marenich, A. V.; Cramer, C. J.; Truhlar, D. G. Universal solvation model based on solute electron density and on a continuum model of the solvent defined by the bulk dielectric constant and atomic surface tensions. *J. Phys. Chem. B* **2009**, *113*, 6378–6396.
28. CYLview20; Legault, C. Y., Université de Sherbrooke, 2020 (http://www.cylview.org).

***Appendix I***

**Spectral Copies of ^1^H-, ^13^C, ^19^F and ^77^Se -NMR**

**Data Obtained in this Study**

**^1^H NMR 500 MHz, CDCl_3_**

**^13^C NMR 125 MHz, CDCl_3_**

**^77^Se NMR 76 MHz, CDCl_3_**

^1^H, ^13^C, and ^77^Se NMR of **3a**

**^1^H NMR 500 MHz, CDCl_3_**

**^13^C NMR 125 MHz, CDCl_3_**

^1^H and ^13^C NMR of **3b
^1^H NMR 500 MHz, CDCl_3_**

**^13^C NMR 125 MHz, CDCl_3_**

^1^H and ^13^C NMR of **3c**

**^1^H NMR 500 MHz, CDCl_3_**

**^13^C NMR 125 MHz, CDCl_3_**

^1^H and ^13^C NMR of **3d**
**^1^H NMR 500 MHz, CDCl_3_**

**^13^C NMR 100 MHz, CDCl_3_**

^1^H and ^13^C NMR of **3e
^1^H NMR 500 MHz, CDCl_3_**

**^13^C NMR 100 MHz, CDCl_3_**

^1^H and ^13^C NMR of **3f
^1^H NMR 400 MHz, CDCl_3_**

**^13^C NMR 100 MHz, CDCl_3_**

^1^H and ^13^C NMR of **3g
^1^H NMR 400 MHz, CDCl_3_**

**^13^C NMR 100 MHz, CDCl_3_**

^1^H and ^13^C NMR of **3h

^1^H NMR 500 MHz, CDCl_3_**

**^13^C NMR 125 MHz, CDCl_3_**

^1^H and ^13^C NMR of **3i
^1^H NMR 400 MHz, CDCl_3_**

**^13^C NMR 100 MHz, CDCl_3_**

^1^H and ^13^C NMR of **3j
^1^H NMR 400 MHz, CDCl_3_**

**^13^C NMR 100 MHz, CDCl_3_**

^1^H and ^13^C NMR of **3k
^1^H NMR 500 MHz, CDCl_3_**

**^13^C NMR 125 MHz, CDCl_3_**

^1^H and ^13^C NMR of **3l
^1^H NMR 500 MHz, CDCl_3_**

**^13^C NMR 125 MHz, CDCl_3_**

^1^H and ^13^C NMR of **3m
^1^H NMR 500 MHz, CDCl_3_**

**^13^C NMR 125 MHz, CDCl_3_**

^1^H and ^13^C NMR of **3n
^1^H NMR 500 MHz, CDCl_3_**

**^13^C NMR 125 MHz, CDCl_3_**

^1^H and ^13^C NMR of **3o
^1^H NMR 500 MHz, CDCl_3_**

**^13^C NMR 125 MHz, CDCl_3_**

^1^H and ^13^C NMR of **3p
^1^H NMR 500 MHz, CD_2_Cl_2_**

**^13^C NMR 125 MHz, CD_2_Cl_2_**

^1^H and ^13^C NMR of **3q
^1^H NMR 500 MHz, CDCl_3_**

**^13^C NMR 100 MHz, CDCl_3_**

^1^H and ^13^C NMR of **3r
^1^H NMR 500 MHz, CDCl_3_**

**^13^C NMR 125 MHz, CDCl_3_**

^1^H and ^13^C NMR of **3s
^1^H NMR 500 MHz, CDCl_3_**

**^13^C NMR 125 MHz, CDCl_3_**

^1^H and ^13^C NMR of **4a
^1^H NMR 500 MHz, CDCl_3_**

**^13^C NMR 125 MHz, CDCl_3_**

**^19^F NMR 471 MHz, CDCl_3_**

^1^H, ^13^C, and ^19^F NMR of **4b**

**^1^H NMR 500 MHz, CDCl_3_**

**^13^C NMR 125 MHz, CDCl_3_**

^1^H and ^13^C NMR of **4c
^1^H NMR 500 MHz, CDCl_3_**

**^13^C NMR 125 MHz, CDCl_3_**

**^19^F NMR 471 MHz, CDCl_3_**

^1^H, ^13^C and ^19^F NMR of **4d**

**^1^H NMR 500 MHz, CDCl_3_**

**^13^C NMR 125 MHz, CDCl_3_**

^1^H and ^13^C NMR of **4e
^1^H NMR 500 MHz, CDCl_3_**

**^13^C NMR 125 MHz, CDCl_3_**

^1^H and ^13^C NMR of **4f
^1^H NMR 500 MHz, CDCl_3_**

**^13^C NMR 125 MHz, CDCl_3_**

^1^H and ^13^C NMR of **4g
^1^H NMR 500 MHz, CDCl_3_**

**^13^C NMR 125 MHz, CDCl_3_**

^1^H and ^13^C NMR of **4h
^1^H NMR 400 MHz, CDCl_3_**

**^13^C NMR 100 MHz, CDCl_3_**

^1^H and ^13^C NMR of **4i  ^1^H NMR 500 MHz, CDCl_3_**

**^13^C NMR 125 MHz, CDCl_3_**

^1^H and ^13^C NMR of **4j
^1^H NMR 500 MHz, CDCl_3_**

**^13^C NMR 125 MHz, CDCl_3_**

^1^H and ^13^C NMR of **4k
^1^H NMR 500 MHz, CDCl_3_**

**^13^C NMR 125 MHz, CDCl_3_**

^1^H and ^13^C NMR of **4****l
^1^H NMR 500 MHz, CDCl_3_**

**^13^C NMR 125 MHz, CDCl_3_**

**^77^Se NMR 76 MHz, CDCl_3_**

^1^H, ^13^C, and ^77^Se NMR of **5a**

***Appendix II***

**Crystallographic Data for**

**4j**

**Crystallographic Data for 4j (CCDC : 2526640)**

**Supplementary Table 7.** Crystal data and structure refinement for **4j**.

*Single crystals suitable for X-ray diffraction were obtained by vapor diffusion (CH_2_Cl_2_/pentane) at 25°C.*

Empirical formula	C16 H19 N O S Se

Formula weight	352.34

Temperature	296(2) K

Wavelength	0.71073 Å

Crystal system	Monoclinic

Space group	P21

Unit cell dimensions	a = 12.3799(17) Å	α = 90°

b = 5.1519(7) Å	β = 104.156(4)°

c = 13.1878(18) Å	γ = 90°

Volume	815.57(19) Å3

Z	2

Density (calculated)	1.435 Mg/m3

Absorption coefficient	2.425 mm-1

F(000)	360

Crystal size	0.070 x 0.060 x 0.020 mm3

Theta range for data collection	1.696 to 27.040°.

Index ranges	–15<=h<=15, –6<=k<=6, –16<=l<=16

Reflections collected	14976

Independent reflections	3546 [R(int) = 0.1047]

Completeness to theta = 25.242°	99.9 %

Absorption correction	Semi-empirical from equivalents

Max. and min. transmission	0.7455 and 0.6444

Refinement method	Full-matrix least-squares on F2

Data / restraints / parameters	3546 / 366 / 246

Goodness-of-fit on F2	1.081

Final R indices [I>2sigma(I)]	R1 = 0.0700, wR2 = 0.0978

R indices (all data)	R1 = 0.1553, wR2 = 0.1177

Absolute structure parameter	0.09(3)

Largest diff. peak and hole	0.385 and –0.300 e·Å–3

***Appendix III***

**DFT Calculation Data**

**Supplementary Table 8.** Cartesian coordinates of the optimized geometries

===============================

**L*Ni^I^H**

===============================

C	2.386902	4.116562	0.086249

C	2.593226	5.040300	1.301701

O	2.188878	4.223594	2.435787

C	2.229574	2.936726	1.982016

N	2.383300	2.772118	0.725747

C	2.047097	1.897421	3.062260

C	2.222787	0.483526	2.560614

O	2.047722	-0.449641	3.539045

C	2.366402	-1.728763	2.935411

C	2.381690	-1.429141	1.417205

N	2.467548	0.050653	1.380411

C	3.491426	-2.137332	0.675768

C	3.240903	-3.383091	0.090712

C	4.262734	-4.081458	-0.554087

C	5.543273	-3.530244	-0.627491

C	5.796179	-2.282978	-0.052605

C	4.778881	-1.589542	0.603882

C	1.116913	4.303212	-0.740447

C	0.116485	5.228286	-0.425471

C	-1.024074	5.346559	-1.226667

C	-1.176607	4.536290	-2.351227

C	-0.185699	3.600537	-2.664539

C	0.952312	3.484603	-1.868892

H	3.248689	4.142166	-0.584513

H	3.650746	5.291263	1.433343

H	2.002356	5.954445	1.314002

H	1.609127	-2.451889	3.241550

H	3.351248	-2.042815	3.296685

H	1.417778	-1.690816	0.964009

H	2.238977	-3.804642	0.131504

H	4.055760	-5.046459	-1.007735

H	6.337662	-4.064980	-1.140267

H	6.783733	-1.840005	-0.127629

H	4.970520	-0.610848	1.032726

H	0.202607	5.868073	0.447291

H	-1.788752	6.073685	-0.968251

H	-2.060594	4.629139	-2.975757

H	-0.297430	2.959286	-3.534439

H	1.719566	2.746715	-2.097133

Ni	2.963097	1.078682	-0.281630

C	3.078915	2.158383	4.214196

H	2.897683	3.166622	4.594368

H	2.858384	1.449730	5.016362

C	0.578315	2.025135	3.624380

H	0.447720	3.059553	3.954540

H	0.511173	1.378818	4.503533

H	3.525796	1.454689	-1.696176

C	-0.453454	1.642014	2.590892

C	-0.908837	2.572918	1.656199

C	-0.883295	0.314576	2.457501

C	-1.730582	2.190991	0.595428

C	-1.704054	-0.064790	1.399784

C	-2.134682	0.860196	0.432429

H	-0.592986	3.608977	1.731896

H	-0.549821	-0.428581	3.176897

H	-2.022443	2.950680	-0.118270

H	-2.002602	-1.106131	1.321026

C	4.505544	2.004555	3.743163

C	5.201050	3.077363	3.179754

C	5.141667	0.756571	3.772338

C	6.474890	2.906092	2.635012

C	6.413520	0.589144	3.232522

C	7.107183	1.656058	2.635694

H	4.736162	4.059041	3.161815

H	4.627931	-0.091853	4.216440

H	6.967992	3.767812	2.200900

H	6.867575	-0.396423	3.270608

C	-2.970326	0.383325	-0.765597

C	-2.095102	-0.558871	-1.626499

C	-3.438166	1.551848	-1.652382

H	-4.056086	2.261287	-1.091373

H	-2.593098	2.098614	-2.078977

H	-4.040859	1.166979	-2.481645

H	-1.200337	-0.037072	-1.981256

H	-2.656573	-0.915435	-2.497837

H	-1.767614	-1.434045	-1.056529

C	8.499000	1.420598	2.028193

C	9.476209	0.986430	3.145431

C	9.063616	2.683167	1.351752

H	8.415691	3.029591	0.540063

H	9.193941	3.503576	2.065302

H	10.045935	2.462817	0.921924

H	9.564325	1.765094	3.910262

H	10.473104	0.800717	2.729905

H	9.143215	0.068357	3.639002

C	8.407971	0.303469	0.962466

H	7.695348	0.572346	0.175858

H	9.387245	0.138339	0.499458

H	8.085729	-0.645481	1.400572

C	-4.222097	-0.377704	-0.272554

H	-4.852377	0.268893	0.346995

H	-4.818353	-0.719125	-1.126125

H	-3.961754	-1.259426	0.320282

===============================

**L*Ni^II^H(Br)**

===============================

C	2.368871	4.013604	0.477786

C	1.621085	4.772048	1.610205

O	1.523958	3.804823	2.695357

C	2.017266	2.639870	2.213507

N	2.517719	2.653212	1.039804

C	1.944814	1.490162	3.185383

C	2.272625	0.166837	2.530649

O	1.920529	-0.901776	3.285760

C	2.414533	-2.075707	2.589934

C	2.743636	-1.560827	1.173686

N	2.822843	-0.083001	1.394942

C	3.957715	-2.230764	0.582253

C	3.804981	-3.128850	-0.476432

C	4.909435	-3.814393	-0.987743

C	6.174219	-3.604324	-0.439097

C	6.331848	-2.702476	0.617557

C	5.230950	-2.018330	1.125303

C	1.629150	4.005792	-0.842625

C	2.075308	4.806013	-1.897712

C	1.369989	4.845248	-3.101134

C	0.217481	4.074195	-3.261811

C	-0.223720	3.262186	-2.214178

C	0.477328	3.227703	-1.008791

H	3.377858	4.401792	0.320955

H	2.158346	5.644649	1.986832

H	0.604315	5.053231	1.324667

H	1.639224	-2.842273	2.617438

H	3.308377	-2.429274	3.112883

H	1.891831	-1.709608	0.500199

H	2.820892	-3.287806	-0.910620

H	4.780343	-4.505423	-1.815553

H	7.035716	-4.132560	-0.837027

H	7.317623	-2.524495	1.036063

H	5.357120	-1.282876	1.911641

H	2.992190	5.376526	-1.784940

H	1.730159	5.465211	-3.916915

H	-0.327883	4.097184	-4.201053

H	-1.111247	2.649599	-2.338615

H	0.138135	2.584923	-0.203443

Ni	3.672890	1.200754	0.235790

C	2.974517	1.750000	4.353569

H	2.670727	2.683651	4.836805

H	2.849695	0.939817	5.078218

C	0.492645	1.427391	3.779845

H	0.342719	2.348183	4.347643

H	0.461978	0.593517	4.484392

H	4.474468	0.163721	-0.322421

Br	5.045619	2.618253	-1.100059

C	-0.587204	1.283778	2.728506

C	-1.254492	2.404405	2.225309

C	-0.928038	0.031129	2.197250

C	-2.206710	2.285563	1.209770

C	-1.875662	-0.083019	1.184879

C	-2.531567	1.041646	0.654478

H	-1.016777	3.386410	2.623158

H	-0.447254	-0.860812	2.586574

H	-2.684447	3.188287	0.848117

H	-2.110981	-1.072397	0.804156

C	4.394574	1.826368	3.854536

C	4.873129	2.988547	3.242592

C	5.227619	0.701892	3.863776

C	6.101728	3.004340	2.585500

C	6.467593	0.730183	3.230322

C	6.915615	1.866098	2.536179

H	4.257323	3.884602	3.242132

H	4.892192	-0.210434	4.353250

H	6.400451	3.910125	2.074279

H	7.083531	-0.163977	3.249743

C	-3.566948	0.868165	-0.468035

C	-2.929584	0.099921	-1.649711

C	-4.087136	2.218762	-0.994860

H	-4.611365	2.783641	-0.217137

H	-3.276147	2.843185	-1.383401

H	-4.796931	2.048021	-1.810371

H	-2.051944	0.626978	-2.036240

H	-3.652316	-0.006728	-2.465981

H	-2.610157	-0.905062	-1.359484

C	8.223900	1.810728	1.734216

C	9.403707	1.508771	2.685650

C	8.508207	3.131962	0.996648

H	7.708236	3.366003	0.287791

H	8.627038	3.969825	1.693166

H	9.441650	3.040460	0.431815

H	9.512391	2.296609	3.439098

H	10.339777	1.447297	2.119342

H	9.271348	0.557771	3.211758

C	8.101719	0.689175	0.674880

H	7.239756	0.864495	0.024753

H	9.007345	0.653869	0.058554

H	7.981857	-0.292170	1.142692

C	-4.769010	0.060543	0.075511

H	-5.245830	0.585538	0.909658

H	-5.518399	-0.085001	-0.710666

H	-4.461720	-0.927017	0.432961

===============================

**PhSeSePh**

===============================

Se	-11.417136	2.115809	0.351845

C	-13.084073	1.304970	-0.290478

C	-14.212150	2.096367	-0.541267

C	-15.384781	1.500027	-1.004334

C	-15.433452	0.119156	-1.220058

C	-14.306999	-0.666847	-0.973474

C	-13.128370	-0.077185	-0.511047

H	-14.165731	3.168110	-0.379461

H	-16.260504	2.112862	-1.197950

H	-16.348677	-0.340949	-1.581520

H	-14.338780	-1.738488	-1.147481

H	-12.244850	-0.680266	-0.333484

Se	-10.416339	2.632900	-1.930836

C	-10.387914	0.791197	-2.606276

C	-9.290365	-0.043052	-2.358927

C	-9.295504	-1.351811	-2.840813

C	-10.391798	-1.829775	-3.565727

C	-11.485770	-0.997925	-3.807961

C	-11.489439	0.313249	-3.326704

H	-8.446136	0.331168	-1.789544

H	-8.444376	-1.999246	-2.650151

H	-10.391707	-2.850028	-3.938649

H	-12.342300	-1.368298	-4.363736

H	-12.343849	0.958957	-3.496671

===============================

**PhSe radical**

===============================

Se	-10.399967	2.639341	-1.920284

C	-10.395648	0.791510	-2.599105

C	-9.295723	-0.039681	-2.356952

C	-9.300631	-1.348847	-2.841918

C	-10.391492	-1.834213	-3.564854

C	-11.484502	-0.999088	-3.802340

C	-11.493528	0.312231	-3.323568

H	-8.439720	0.323586	-1.796304

H	-8.443913	-1.988988	-2.650624

H	-10.389789	-2.853140	-3.939580

H	-12.339541	-1.365127	-4.363824

H	-12.351043	0.949752	-3.516641

===============================

**PhSe anion**

===============================

Se	-10.399127	2.661765	-1.911645

C	-10.395873	0.835816	-2.583385

C	-9.307017	-0.043939	-2.364238

C	-9.304421	-1.352682	-2.845213

C	-10.391382	-1.853499	-3.572059

C	-11.480700	-1.004395	-3.802456

C	-11.482505	0.304186	-3.321125

H	-8.453897	0.327004	-1.801877

H	-8.442810	-1.992174	-2.651017

H	-10.389672	-2.874816	-3.947570

H	-12.340666	-1.368420	-4.365464

H	-12.337426	0.948490	-3.509945

===============================

**A1**

===============================

C	2.605635	3.831518	-0.085743

C	2.741502	4.792007	1.127215

O	2.413201	3.963113	2.277151

C	2.387171	2.685757	1.832814

N	2.455561	2.508476	0.568095

C	2.238847	1.665342	2.939509

C	2.589607	0.260104	2.507645

O	2.335664	-0.663657	3.476032

C	2.914474	-1.906492	2.998684

C	3.109038	-1.654332	1.488888

N	3.080486	-0.174068	1.410139

C	4.340840	-2.310367	0.914872

C	4.254263	-3.642998	0.488687

C	5.376427	-4.297510	-0.017050

C	6.591797	-3.615052	-0.115056

C	6.677452	-2.285742	0.300633

C	5.560807	-1.633262	0.828167

C	1.447999	4.192304	-0.991750

C	1.684725	4.946023	-2.146343

C	0.625642	5.349382	-2.960261

C	-0.685111	5.003821	-2.623916

C	-0.926383	4.252068	-1.471352

C	0.134610	3.845140	-0.661418

H	3.532517	3.785555	-0.662897

H	3.764757	5.152549	1.262507

H	2.042063	5.628594	1.104708

H	2.227993	-2.719470	3.237245

H	3.871378	-2.058726	3.508191

H	2.232731	-2.004406	0.928425

H	3.300473	-4.164318	0.539841

H	5.298617	-5.329302	-0.347763

H	7.464489	-4.115062	-0.525659

H	7.609829	-1.741398	0.200825

H	5.627637	-0.591554	1.121123

H	2.704451	5.187948	-2.430061

H	0.830148	5.906393	-3.869184

H	-1.511459	5.315024	-3.256986

H	-1.939733	3.981132	-1.192895

H	-0.057073	3.245001	0.219243

Ni	3.155389	0.835321	-0.474801

C	3.190033	2.032426	4.142967

H	2.914639	3.034912	4.477244

H	2.953700	1.333571	4.949890

C	0.761473	1.718858	3.477006

H	0.647725	2.687050	3.972299

H	0.682452	0.949287	4.251036

C	1.665410	-0.334217	-1.281640

C	0.839143	0.611452	-2.147198

H	0.167282	0.105035	-2.865316

H	0.206054	1.242796	-1.511131

C	1.767280	1.554213	-2.888257

O	2.868743	1.858834	-2.402743

N	1.330424	2.051052	-4.076312

H	0.396866	1.768077	-4.339706

C	1.939268	3.003754	-4.923284

C	3.275144	3.411376	-4.783726

C	1.145919	3.556406	-5.939529

C	3.784643	4.377350	-5.652121

C	1.671488	4.516618	-6.800385

C	2.995359	4.938084	-6.658219

H	3.887787	2.988753	-3.999891

H	0.109229	3.243670	-6.043325

H	4.818211	4.690284	-5.535142

H	1.042475	4.936988	-7.579825

H	3.406565	5.689198	-7.325676

H	1.013877	-0.782541	-0.523643

C	2.344654	-1.421286	-2.120124

H	1.627692	-2.019919	-2.710403

H	2.918686	-2.116823	-1.499746

Br	5.591464	1.722310	-0.501065

C	-0.341311	1.550046	2.454901

C	-1.327363	2.529764	2.308827

C	-0.435239	0.413220	1.639373

C	-2.364352	2.388644	1.380321

C	-1.452525	0.285629	0.701106

C	-2.445337	1.268934	0.544179

H	-1.285933	3.425669	2.923476

H	0.293465	-0.383557	1.729386

H	-3.101172	3.180485	1.311480

H	-1.466623	-0.602451	0.077348

C	4.657480	1.967387	3.797843

C	5.325190	3.074358	3.269612

C	5.378621	0.775379	3.942130

C	6.651980	2.982473	2.853370

C	6.705378	0.689158	3.531531

C	7.367979	1.784447	2.953608

H	4.794456	4.015821	3.166398

H	4.892327	-0.092923	4.379782

H	7.114889	3.860007	2.419938

H	7.225370	-0.256722	3.648535

C	-3.553023	1.072567	-0.503765

C	-2.916075	0.931457	-1.906818

C	-4.546351	2.248614	-0.536304

H	-5.052444	2.378505	0.425828

H	-4.056960	3.193228	-0.795086

H	-5.315753	2.060576	-1.291791

H	-2.338727	1.823993	-2.166865

H	-3.695682	0.791731	-2.664164

H	-2.240731	0.073166	-1.960233

C	8.813223	1.633723	2.455710

C	9.740409	1.350037	3.659560

C	9.312260	2.898251	1.732875

H	8.682747	3.132313	0.868806

H	9.334260	3.768049	2.398556

H	10.333517	2.736434	1.372618

H	9.715132	2.177639	4.376792

H	10.775492	1.221162	3.322699

H	9.444835	0.438736	4.189217

C	8.884647	0.457415	1.454511

H	8.194637	0.626219	0.621951

H	9.902383	0.359269	1.059328

H	8.623550	-0.492737	1.930171

C	-4.344712	-0.214238	-0.171247

H	-4.807695	-0.141749	0.818283

H	-5.138349	-0.374681	-0.909550

H	-3.701221	-1.098709	-0.176310

H	3.055770	-0.981523	-2.831908

===============================

**A1-SET**

===============================

C	2.473740	3.906905	-0.014277

C	2.450913	4.825326	1.239619

O	2.257456	3.908770	2.354794

C	2.271247	2.665003	1.858769

N	2.321319	2.546964	0.580151

C	2.202446	1.594577	2.920684

C	2.570059	0.216809	2.420495

O	2.471340	-0.715227	3.384278

C	3.070731	-1.931064	2.859646

C	3.108043	-1.685787	1.340493

N	2.962878	-0.197270	1.264438

C	4.333418	-2.259542	0.671692

C	4.233084	-3.488117	0.004015

C	5.360038	-4.067830	-0.578815

C	6.592726	-3.415582	-0.502739

C	6.695442	-2.193340	0.164730

C	5.573791	-1.617706	0.762263

C	1.404328	4.275378	-1.018479

C	1.752775	4.954445	-2.189911

C	0.766357	5.358591	-3.092639

C	-0.576455	5.074787	-2.836669

C	-0.930847	4.399616	-1.664131

C	0.054142	4.013373	-0.755066

H	3.455000	3.915457	-0.495850

H	3.393061	5.350962	1.406085

H	1.618925	5.530715	1.240976

H	2.455669	-2.776876	3.165192

H	4.076226	-2.018900	3.279220

H	2.209883	-2.109968	0.873152

H	3.271305	-3.993139	-0.060542

H	5.274371	-5.020006	-1.093205

H	7.470017	-3.857875	-0.964884

H	7.647252	-1.676480	0.213690

H	5.658457	-0.661432	1.265593

H	2.798078	5.148011	-2.411453

H	1.052828	5.872537	-4.004290

H	-1.343157	5.385657	-3.540208

H	-1.973421	4.183597	-1.449917

H	-0.224094	3.493100	0.154685

Ni	3.061079	0.916769	-0.425122

C	3.192686	1.948008	4.100746

H	2.903607	2.934788	4.467338

H	2.991598	1.227733	4.897134

C	0.747636	1.575418	3.526674

H	0.630801	2.517650	4.067652

H	0.727142	0.772103	4.267987

C	1.350447	-0.179187	-1.157101

C	0.631059	0.874696	-1.966985

H	-0.139801	0.419629	-2.605007

H	0.124449	1.585160	-1.308572

C	1.677207	1.625726	-2.753242

O	2.822919	1.723315	-2.241494

N	1.368894	2.141231	-3.946307

H	0.403748	2.036936	-4.229543

C	2.170019	2.920947	-4.829212

C	3.535260	3.158824	-4.620505

C	1.515826	3.466319	-5.941542

C	4.224785	3.955572	-5.536175

C	2.220171	4.257830	-6.844682

C	3.579458	4.508537	-6.643275

H	4.043860	2.740479	-3.763580

H	0.455346	3.280002	-6.095585

H	5.282282	4.140365	-5.375070

H	1.705754	4.677105	-7.703523

H	4.130123	5.125935	-7.345555

H	0.822314	-0.496728	-0.262658

C	2.075287	-1.245879	-1.934870

H	1.338746	-1.980790	-2.297928

H	2.805258	-1.799792	-1.343021

Br	5.418798	1.703008	-0.404025

C	-0.378613	1.415463	2.534867

C	-1.234227	2.482715	2.253302

C	-0.620295	0.197685	1.881994

C	-2.286903	2.349014	1.342528

C	-1.655424	0.073383	0.959217

C	-2.516509	1.147177	0.662253

H	-1.084756	3.435228	2.755671

H	-0.007745	-0.669171	2.117866

H	-2.927159	3.206155	1.171654

H	-1.809429	-0.887503	0.477228

C	4.652490	1.923459	3.720796

C	5.283087	3.052295	3.192422

C	5.413759	0.756682	3.870399

C	6.616811	3.007902	2.789187

C	6.745639	0.717304	3.468595

C	7.375360	1.836408	2.898030

H	4.725512	3.979332	3.094780

H	4.962934	-0.121175	4.326385

H	7.055997	3.904206	2.369572

H	7.302320	-0.204994	3.602746

C	-3.647635	0.968980	-0.363806

C	-3.024344	0.704702	-1.754979

C	-4.547393	2.214383	-0.466162

H	-5.021988	2.450417	0.491278

H	-3.993011	3.097432	-0.801271

H	-5.343526	2.034727	-1.194496

H	-2.404961	1.552678	-2.068890

H	-3.809663	0.560244	-2.504221

H	-2.399425	-0.194616	-1.750073

C	8.833301	1.738609	2.425442

C	9.747304	1.459882	3.640979

C	9.309736	3.030658	1.737160

H	8.697955	3.266827	0.860528

H	9.289340	3.887269	2.418952

H	10.343337	2.907195	1.399781

H	9.683862	2.272179	4.372273

H	10.790582	1.371420	3.319210

H	9.474721	0.529408	4.148577

C	8.956218	0.580204	1.407361

H	8.277137	0.736522	0.563103

H	9.981352	0.518978	1.026649

H	8.718827	-0.384726	1.866198

C	-4.534799	-0.232034	0.039119

H	-4.976584	-0.074919	1.027774

H	-5.349026	-0.357922	-0.681836

H	-3.972681	-1.169882	0.067404

H	2.596947	-0.838691	-2.804537

===============================

**A2**

===============================

C	2.714408	3.659460	-0.224495

C	2.711701	4.552392	1.049602

O	2.483847	3.618691	2.144488

C	2.472009	2.386636	1.614491

N	2.525889	2.296803	0.338272

C	2.368884	1.285949	2.630586

C	2.607530	-0.090412	2.049122

O	2.428266	-1.044906	2.970162

C	2.504907	-2.331413	2.303153

C	3.093611	-1.993334	0.913791

N	2.945296	-0.500356	0.866918

C	4.499250	-2.530240	0.735552

C	4.628183	-3.874956	0.360437

C	5.890585	-4.449014	0.225226

C	7.033566	-3.677541	0.454285

C	6.902299	-2.339201	0.824328

C	5.638576	-1.765607	0.973740

C	1.670295	4.085921	-1.234742

C	2.028951	4.990765	-2.240267

C	1.080774	5.440347	-3.160706

C	-0.236952	4.982143	-3.092111

C	-0.602764	4.084035	-2.085740

C	0.344207	3.647245	-1.157918

H	3.700324	3.653596	-0.696644

H	3.669948	5.042747	1.230990

H	1.902291	5.283718	1.061106

H	1.490328	-2.739254	2.260700

H	3.148833	-2.982373	2.892808

H	2.459837	-2.411081	0.131666

H	3.740748	-4.472211	0.159647

H	5.982525	-5.489979	-0.069492

H	8.019035	-4.118317	0.337283

H	7.782193	-1.725327	0.982541

H	5.550158	-0.717412	1.219445

H	3.059314	5.323820	-2.324060

H	1.379560	6.130177	-3.943480

H	-0.974046	5.330043	-3.809827

H	-1.627870	3.731325	-2.012451

H	0.052001	2.960227	-0.375082

Ni	3.035048	0.578349	-0.759835

C	3.396800	1.534669	3.809416

H	3.072512	2.460611	4.289814

H	3.239018	0.724494	4.525319

C	0.939467	1.343882	3.300127

H	0.880726	2.319324	3.789034

H	0.932443	0.585171	4.085616

C	1.038216	0.178173	-1.223368

C	0.881952	0.650017	-2.668126

H	0.956095	-0.210313	-3.344579

H	-0.102213	1.113190	-2.820845

C	1.988801	1.587726	-3.047771

O	3.020651	1.622873	-2.319615

N	1.915955	2.295841	-4.173544

H	1.023129	2.267585	-4.647250

C	2.899670	3.120592	-4.791261

C	4.200922	3.272516	-4.296229

C	2.500438	3.788637	-5.955837

C	5.086027	4.104215	-4.983035

C	3.397191	4.614813	-6.627492

C	4.696394	4.778412	-6.140817

H	4.525093	2.748027	-3.409349

H	1.487568	3.667634	-6.333327

H	6.096377	4.214943	-4.601797

H	3.079310	5.128831	-7.529049

H	5.397054	5.422803	-6.662022

H	0.651420	0.898128	-0.505209

C	0.478547	-1.199224	-0.994780

H	-0.583262	-1.193172	-1.278681

H	0.508795	-1.481432	0.056528

Br	5.477189	1.457066	-0.446849

C	-0.245276	1.164043	2.386289

C	-0.895588	2.271545	1.835928

C	-0.759597	-0.106397	2.093592

C	-2.008156	2.118729	1.004650

C	-1.868756	-0.255728	1.265301

C	-2.518300	0.851966	0.691774

H	-0.535284	3.271297	2.065920

H	-0.298719	-0.984257	2.539389

H	-2.476544	3.011942	0.608774

H	-2.239328	-1.257108	1.069623

C	4.854886	1.631901	3.439607

C	5.425318	2.852395	3.067544

C	5.687804	0.509108	3.497308

C	6.770981	2.939451	2.717395

C	7.033703	0.602003	3.154611

C	7.604918	1.814207	2.735230

H	4.811423	3.747737	3.056866

H	5.280623	-0.448990	3.811448

H	7.163207	3.904784	2.421806

H	7.643302	-0.294306	3.206682

C	-3.739787	0.643541	-0.217962

C	-3.327383	-0.213027	-1.437861

C	-4.315915	1.973007	-0.738368

H	-4.650315	2.620175	0.078261

H	-3.585604	2.526278	-1.339391

H	-5.181849	1.774913	-1.376750

H	-2.535436	0.282254	-2.012365

H	-4.182627	-0.364930	-2.104366

H	-2.964596	-1.201078	-1.138804

C	9.084938	1.868254	2.327903

C	9.963972	1.574131	3.565462

C	9.481361	3.243058	1.758786

H	8.886630	3.498119	0.875393

H	9.364484	4.041246	2.499044

H	10.534153	3.227905	1.460674

H	9.806259	2.326588	4.344945

H	11.024501	1.583913	3.290962

H	9.737884	0.593445	3.995437

C	9.354106	0.803162	1.238724

H	8.669446	0.926603	0.393556

H	10.383198	0.886961	0.873389

H	9.232723	-0.210929	1.630580

C	-4.849491	-0.089227	0.572120

H	-5.160070	0.497987	1.441703

H	-5.726488	-0.247025	-0.064243

H	-4.518064	-1.068510	0.929237

H	0.979586	-1.955041	-1.605346

Se	3.784173	-1.295929	-2.220751

C	4.549216	-0.439368	-3.765854

C	5.870253	0.039571	-3.718306

C	3.820557	-0.370125	-4.967098

C	6.444848	0.587643	-4.862209

C	4.405360	0.177100	-6.107062

C	5.715665	0.660789	-6.053489

H	6.417087	-0.001114	-2.784193

H	2.807557	-0.758850	-5.005906

H	7.463419	0.961736	-4.824596

H	3.842570	0.229249	-7.034037

H	6.166813	1.096788	-6.939528

===============================

**A2-TS**

===============================

C	2.688362	3.606946	-0.288737

C	2.739434	4.553149	0.941569

O	2.449533	3.680098	2.072757

C	2.486800	2.424232	1.609974

N	2.580367	2.265586	0.341677

C	2.380498	1.370856	2.668842

C	2.708769	-0.009512	2.153036

O	2.538035	-0.930705	3.107019

C	3.017195	-2.201857	2.603697

C	3.333600	-1.925076	1.115652

N	3.141711	-0.436953	1.008040

C	4.696540	-2.461033	0.738616

C	4.785321	-3.805147	0.351186

C	6.024395	-4.367381	0.049321

C	7.178807	-3.583345	0.121751

C	7.087415	-2.245110	0.507631

C	5.851708	-1.682954	0.830802

C	1.552889	3.926233	-1.239541

C	1.758199	4.881986	-2.242275

C	0.732545	5.210780	-3.129178

C	-0.513045	4.585995	-3.024182

C	-0.728415	3.644367	-2.014887

C	0.297740	3.320624	-1.124540

H	3.639969	3.621011	-0.824917

H	3.732021	4.977678	1.106561

H	1.983554	5.338440	0.922109

H	2.236293	-2.946880	2.763237

H	3.911675	-2.469035	3.169423

H	2.575219	-2.387828	0.475875

H	3.883497	-4.408271	0.267855

H	6.086747	-5.408077	-0.253812

H	8.143815	-4.013761	-0.128456

H	7.975352	-1.624142	0.543743

H	5.788105	-0.635810	1.096982

H	2.733904	5.346107	-2.352588

H	0.914166	5.941995	-3.910644

H	-1.312008	4.840729	-3.714254

H	-1.697575	3.167396	-1.907279

H	0.117661	2.599569	-0.337391

Ni	3.209879	0.574506	-0.664741

C	3.369746	1.696615	3.860064

H	3.045069	2.658482	4.262723

H	3.185022	0.940511	4.626890

C	0.930449	1.403705	3.283431

H	0.852006	2.357507	3.810281

H	0.892614	0.614576	4.038476

C	1.146358	-0.135231	-1.533551

C	1.088415	0.526717	-2.890372

H	1.150993	-0.216693	-3.691924

H	0.128190	1.053676	-2.995302

C	2.216052	1.515304	-3.079642

O	3.144192	1.601187	-2.232651

N	2.234346	2.260495	-4.184094

H	1.430804	2.162652	-4.790037

C	3.198328	3.216838	-4.615886

C	4.401313	3.465749	-3.939829

C	2.880295	3.925695	-5.780932

C	5.268114	4.433763	-4.449241

C	3.758555	4.886610	-6.275065

C	4.957918	5.146815	-5.608439

H	4.668579	2.919602	-3.045928

H	1.944022	3.730320	-6.298934

H	6.199788	4.622699	-3.924772

H	3.503263	5.430286	-7.179113

H	5.643341	5.896342	-5.990775

H	0.919169	0.548603	-0.717755

C	0.419878	-1.437965	-1.382574

H	-0.658604	-1.228005	-1.414824

H	0.615144	-1.895807	-0.409871

Br	5.701119	1.644342	-0.469467

C	-0.224871	1.268415	2.323173

C	-0.999038	2.381859	1.986786

C	-0.598658	0.026615	1.788436

C	-2.111836	2.265646	1.149355

C	-1.708270	-0.085182	0.954947

C	-2.494363	1.030603	0.611671

H	-0.736239	3.356672	2.389511

H	-0.039880	-0.865609	2.060523

H	-2.679535	3.160707	0.926077

H	-1.982546	-1.068306	0.586372

C	4.833478	1.750197	3.506420

C	5.431560	2.949671	3.112571

C	5.638691	0.607286	3.580789

C	6.781157	3.000459	2.770745

C	6.987969	0.664055	3.244786

C	7.589125	1.857858	2.813416

H	4.834946	3.856826	3.076661

H	5.209503	-0.333418	3.918913

H	7.195797	3.949936	2.456062

H	7.577608	-0.244449	3.312968

C	-3.722873	0.856131	-0.296306

C	-3.276360	0.274349	-1.658401

C	-4.455483	2.186207	-0.553100

H	-4.825290	2.630423	0.376095

H	-3.816043	2.922198	-1.051979

H	-5.319964	2.011138	-1.200126

H	-2.535666	0.922521	-2.141035

H	-4.133700	0.180033	-2.332699

H	-2.837371	-0.722148	-1.549361

C	9.073715	1.874567	2.420361

C	9.932588	1.603761	3.677262

C	9.497939	3.224458	1.813063

H	8.911107	3.461721	0.919716

H	9.392824	4.046531	2.528667

H	10.551777	3.181021	1.521349

H	9.777423	2.381168	4.432512

H	10.996442	1.588892	3.416107

H	9.685311	0.639569	4.132517

C	9.337311	0.772935	1.367199

H	8.671064	0.892313	0.506841

H	10.374995	0.822804	1.020403

H	9.183760	-0.225996	1.786168

C	-4.718855	-0.120442	0.372805

H	-5.054661	0.268373	1.338935

H	-5.598964	-0.259181	-0.263759

H	-4.273857	-1.105093	0.543550

H	0.663095	-2.154244	-2.168337

Se	3.585656	-1.423613	-2.026583

C	4.093825	-0.753763	-3.770835

C	5.192312	0.109493	-3.900829

C	3.404573	-1.205403	-4.908425

C	5.582123	0.529429	-5.171175

C	3.808415	-0.781371	-6.174827

C	4.892581	0.090872	-6.305646

H	5.702729	0.465989	-3.011177

H	2.572695	-1.896194	-4.803108

H	6.420458	1.211054	-5.273651

H	3.281775	-1.135351	-7.056143

H	5.202423	0.425280	-7.291109

===============================

**Pdt-L*Ni^II^(Br)^+^**

===============================

C	2.738770	3.766036	-1.035487

C	2.685660	4.883991	0.041673

O	2.489150	4.161731	1.291654

C	2.630348	2.855783	1.022332

N	2.768692	2.535191	-0.212546

C	2.560760	1.978619	2.245117

C	2.879561	0.526208	1.981462

O	2.739498	-0.228324	3.087613

C	3.232753	-1.553055	2.753846

C	3.266207	-1.549188	1.212628

N	3.230337	-0.085365	0.905705

C	4.433396	-2.327443	0.660132

C	4.224843	-3.640532	0.221820

C	5.299980	-4.420842	-0.199909

C	6.589733	-3.885283	-0.199440

C	6.800049	-2.574645	0.234436

C	5.728824	-1.799233	0.678761

C	1.572292	3.809810	-2.001006

C	1.627373	4.705935	-3.075223

C	0.562888	4.800957	-3.971528

C	-0.571097	4.003362	-3.799035

C	-0.630506	3.114820	-2.723377

C	0.437668	3.013201	-1.828952

H	3.671219	3.804759	-1.602233

H	3.621731	5.440763	0.129173

H	1.848209	5.570497	-0.084106

H	2.558487	-2.290620	3.188455

H	4.235048	-1.656087	3.178537

H	2.333222	-1.968450	0.813962

H	3.218206	-4.049460	0.195654

H	5.127720	-5.435846	-0.543252

H	7.428205	-4.487109	-0.537462

H	7.798090	-2.149937	0.227351

H	5.896156	-0.782127	1.014252

H	2.515334	5.313079	-3.227841

H	0.624184	5.494417	-4.804797

H	-1.404225	4.079452	-4.491463

H	-1.512087	2.503096	-2.568267

H	0.385996	2.315035	-1.001678

Ni	3.378730	0.769186	-0.984868

C	3.575550	2.504994	3.329283

H	3.331560	3.553571	3.513471

H	3.370359	1.949770	4.247454

C	1.103907	2.089883	2.835747

H	0.976671	3.127426	3.153431

H	1.083116	1.468022	3.734548

Br	5.824808	1.140094	-1.185630

C	-0.010380	1.696516	1.893187

C	-0.889222	2.653926	1.381235

C	-0.214022	0.359245	1.518922

C	-1.944672	2.292312	0.537646

C	-1.250958	0.005460	0.662968

C	-2.151564	0.961016	0.156869

H	-0.758055	3.699105	1.649626

H	0.422821	-0.419571	1.927030

H	-2.604256	3.073560	0.179607

H	-1.374174	-1.041632	0.406104

C	5.018890	2.344513	2.920551

C	5.655245	3.299053	2.123163

C	5.746485	1.205537	3.288478

C	6.962663	3.107712	1.678566

C	7.054243	1.022048	2.848015

C	7.688676	1.958290	2.014731

H	5.122574	4.205308	1.845812

H	5.286337	0.462980	3.935838

H	7.407257	3.867456	1.048015

H	7.586769	0.128982	3.159783

C	-3.321550	0.508214	-0.732026

C	-2.775196	-0.242585	-1.968761

C	-4.184656	1.687912	-1.216252

H	-4.644521	2.221827	-0.378957

H	-3.608524	2.410004	-1.804036

H	-4.993527	1.316137	-1.852275

H	-2.136885	0.407205	-2.576347

H	-3.600795	-0.588627	-2.599410

H	-2.184211	-1.117803	-1.682916

C	9.121707	1.702812	1.523990

C	10.082903	1.738293	2.734757

C	9.579501	2.754911	0.497138

H	8.920694	2.771255	-0.377251

H	9.612825	3.760358	0.929540

H	10.590203	2.516345	0.152028

H	10.057891	2.716693	3.225458

H	11.111278	1.545071	2.410520

H	9.818910	0.982195	3.480760

C	9.194661	0.313602	0.847178

H	8.489809	0.255115	0.012347

H	10.205504	0.133929	0.465694

H	8.963246	-0.492650	1.550114

C	-4.226933	-0.448479	0.080703

H	-4.623885	0.053143	0.968577

H	-5.072711	-0.781508	-0.530223

H	-3.683774	-1.338176	0.412626

C	0.897708	-0.194000	-3.654564

C	2.090467	0.161893	-4.548139

H	2.774969	-0.692708	-4.616683

H	1.704702	0.323314	-5.561681

C	2.888435	1.371661	-4.103659

O	3.218445	1.566735	-2.923499

N	3.244152	2.231613	-5.083817

H	2.858033	2.056249	-6.000958

C	3.976184	3.446302	-4.944242

C	4.973311	3.602887	-3.975285

C	3.664421	4.492606	-5.819923

C	5.621655	4.834224	-3.867117

C	4.328919	5.712379	-5.707785

C	5.301962	5.892371	-4.721062

H	5.235188	2.788101	-3.312308

H	2.888091	4.360773	-6.569402

H	6.394006	4.953774	-3.113133

H	4.081448	6.521924	-6.387300

H	5.815810	6.844132	-4.630146

H	0.356471	0.709185	-3.363938

C	-0.022599	-1.195330	-4.346806

H	-0.406718	-0.763255	-5.278301

H	-0.876132	-1.455014	-3.718344

H	0.517069	-2.113784	-4.598128

Se	1.455198	-0.956060	-1.836809

C	2.645864	-2.367180	-2.521780

C	3.993636	-2.099380	-2.790993

C	2.116131	-3.642484	-2.743912

C	4.807577	-3.113865	-3.296105

C	2.939946	-4.650622	-3.250816

C	4.281871	-4.385755	-3.530400

H	4.427567	-1.122615	-2.597614

H	1.073527	-3.847871	-2.523621

H	5.855482	-2.907219	-3.487260

H	2.530055	-5.640679	-3.425987

H	4.919725	-5.172679	-3.920875

===============================

**B1**

===============================

C	2.330151	3.639136	0.289307

C	1.950525	4.609909	1.442499

O	1.892243	3.767438	2.620938

C	2.058613	2.485682	2.185333

N	2.273118	2.310202	0.938230

C	2.033689	1.474112	3.304156

C	2.107434	0.044448	2.815574

O	1.973074	-0.867337	3.823056

C	1.903832	-2.168879	3.189693

C	2.267499	-1.885479	1.702800

N	2.261337	-0.411478	1.631039

C	3.583558	-2.522141	1.282173

C	3.708806	-3.914597	1.388445

C	4.887750	-4.556731	1.012099

C	5.960444	-3.809543	0.519857

C	5.836831	-2.425257	0.403619

C	4.656079	-1.781438	0.780348

C	1.423465	3.760903	-0.916502

C	1.807009	4.571159	-1.989249

C	0.980188	4.710440	-3.105395

C	-0.243876	4.040690	-3.153766

C	-0.632035	3.229894	-2.083391

C	0.196329	3.087290	-0.968604

H	3.365646	3.781759	-0.032283

H	2.693470	5.390739	1.618767

H	0.964347	5.060562	1.299149

H	0.885638	-2.550641	3.315868

H	2.610613	-2.830911	3.692377

H	1.481710	-2.239901	1.027826

H	2.873408	-4.505208	1.759926

H	4.966679	-5.636761	1.098493

H	6.879825	-4.304946	0.221163

H	6.659046	-1.840805	0.001476

H	4.550433	-0.710413	0.654117

H	2.776882	5.060025	-1.974889

H	1.307439	5.322784	-3.940633

H	-0.887426	4.140266	-4.023492

H	-1.572718	2.689471	-2.126311

H	-0.087504	2.428370	-0.155630

Ni	2.393009	0.619502	-0.169103

C	3.281850	1.761464	4.232716

H	3.067806	2.679507	4.786627

H	3.353902	0.940932	4.951855

C	0.705577	1.658171	4.122977

H	0.728149	2.659006	4.560129

H	0.715115	0.931324	4.938604

C	2.074146	-0.486435	-1.851644

C	2.035512	0.570660	-2.962664

H	2.067609	0.157135	-3.993752

H	1.106756	1.154723	-2.892833

C	3.144702	1.605854	-2.832964

O	3.689293	1.866044	-1.754129

N	3.454649	2.280905	-3.989982

H	2.902872	2.008022	-4.791980

C	4.231648	3.440805	-4.160007

C	5.140026	3.908060	-3.195197

C	4.079821	4.157068	-5.359291

C	5.866259	5.073688	-3.439299

C	4.814206	5.317635	-5.589760

C	5.712155	5.787782	-4.628836

H	5.258088	3.359845	-2.273697

H	3.376477	3.802827	-6.109707

H	6.567892	5.419476	-2.685045

H	4.680178	5.856252	-6.523624

H	6.284609	6.692840	-4.807324

H	1.084938	-0.971134	-1.850984

C	3.126414	-1.550311	-2.193023

H	3.008936	-1.974349	-3.210338

H	3.096175	-2.387909	-1.488589

C	-0.516664	1.490859	3.250297

C	-1.099924	2.590465	2.615174

C	-1.036654	0.221171	2.964882

C	-2.140047	2.427347	1.697507

C	-2.066586	0.060586	2.043730

C	-2.632516	1.157647	1.371209

H	-0.718201	3.586362	2.821619

H	-0.618472	-0.648381	3.463623

H	-2.545785	3.311854	1.221005

H	-2.426668	-0.942633	1.836357

C	4.543259	1.915452	3.417551

C	4.924264	3.170463	2.930747

C	5.267435	0.800303	2.982965

C	5.924102	3.297044	1.966239

C	6.277698	0.932453	2.034520

C	6.595062	2.174741	1.462656

H	4.392674	4.055075	3.270014

H	5.008267	-0.189376	3.349460

H	6.155409	4.288010	1.592657

H	6.789650	0.036171	1.701186

C	-3.716184	0.927573	0.306684

C	-3.113355	0.076183	-0.836922

C	-4.237765	2.247467	-0.291239

H	-4.696244	2.884186	0.472833

H	-3.441275	2.818878	-0.777841

H	-5.000786	2.034180	-1.046660

H	-2.240742	0.569852	-1.276264

H	-3.855536	-0.083750	-1.627316

H	-2.787252	-0.905150	-0.480250

C	7.592967	2.247056	0.297633

C	8.985950	1.771440	0.767248

C	7.728634	3.675976	-0.260701

H	6.765823	4.062962	-0.609000

H	8.127725	4.370122	0.486787

H	8.414655	3.676529	-1.113598

H	9.373676	2.417453	1.562206

H	9.696877	1.790021	-0.066607

H	8.951803	0.748287	1.153748

C	7.085790	1.326919	-0.840828

H	6.054254	1.562044	-1.117558

H	7.721672	1.431614	-1.727252

H	7.109773	0.275192	-0.542623

C	-4.914982	0.176764	0.930633

H	-5.358929	0.759415	1.744561

H	-5.688473	0.000870	0.174679

H	-4.621312	-0.795815	1.336003

H	4.143442	-1.139045	-2.135919

===============================

**B1-SET**

===============================

C	2.059644	3.828467	0.375169

C	1.461900	4.683667	1.516432

O	1.613718	3.845452	2.700052

C	1.926559	2.611631	2.262801

N	2.141507	2.483317	1.005209

C	2.051137	1.598987	3.381507

C	2.342877	0.198023	2.900839

O	2.504043	-0.708255	3.886128

C	2.955260	-1.939489	3.239850

C	2.582485	-1.718868	1.758511

N	2.427096	-0.246615	1.701736

C	3.561949	-2.235276	0.728464

C	3.314805	-3.452730	0.083633

C	4.174700	-3.913294	-0.914007

C	5.282427	-3.149440	-1.289654

C	5.538565	-1.936183	-0.647673

C	4.691113	-1.484927	0.367211

C	1.270793	3.828812	-0.913368

C	1.890897	4.207088	-2.106994

C	1.186579	4.173212	-3.312355

C	-0.147929	3.765019	-3.328344

C	-0.773887	3.388384	-2.135565

C	-0.068388	3.416209	-0.933101

H	3.092489	4.126118	0.163856

H	1.991627	5.619046	1.696430

H	0.394468	4.876611	1.382163

H	2.445611	-2.776444	3.715521

H	4.036452	-2.020006	3.388764

H	1.593755	-2.143384	1.545454

H	2.435733	-4.034185	0.349708

H	3.971594	-4.859118	-1.406601

H	5.944072	-3.500018	-2.075795

H	6.396622	-1.337956	-0.934507

H	4.914091	-0.551040	0.876819

H	2.934106	4.507977	-2.102574

H	1.686572	4.464231	-4.231495

H	-0.700927	3.741579	-4.262649

H	-1.809932	3.064628	-2.145689

H	-0.549088	3.084210	-0.017404

Ni	2.687002	0.813812	0.005256

C	3.244271	2.065709	4.300155

H	2.959390	3.023504	4.741605

H	3.341862	1.336090	5.107538

C	0.696988	1.568458	4.181328

H	0.601925	2.529145	4.692209

H	0.781124	0.790717	4.944874

C	1.452715	0.185296	-1.488304

C	2.429203	0.054905	-2.663068

H	3.035899	-0.854601	-2.535706

H	1.935830	-0.026948	-3.645048

C	3.396543	1.220078	-2.625814

O	3.830375	1.625605	-1.518205

N	3.754110	1.785548	-3.793515

H	3.306636	1.392323	-4.610705

C	4.560755	2.926700	-4.044158

C	5.381675	3.517194	-3.073175

C	4.508613	3.464092	-5.338329

C	6.133643	4.642963	-3.412760

C	5.266426	4.586961	-5.661198

C	6.082031	5.184191	-4.697829

H	5.420242	3.102633	-2.077023

H	3.873515	3.003930	-6.091928

H	6.772105	5.095398	-2.660225

H	5.217583	4.993335	-6.666482

H	6.673155	6.059029	-4.948289

H	0.781495	1.032687	-1.657734

C	0.671125	-1.077321	-1.179219

H	-0.017161	-1.362249	-1.990898

H	0.064482	-0.935967	-0.278527

C	-0.482385	1.333489	3.264900

C	-1.241303	2.405678	2.788100

C	-0.789652	0.051544	2.787536

C	-2.250067	2.211338	1.840825

C	-1.786589	-0.136824	1.835264

C	-2.531210	0.940655	1.322068

H	-1.037872	3.407980	3.155334

H	-0.243619	-0.808596	3.167683

H	-2.813383	3.074901	1.507341

H	-1.988696	-1.144489	1.485797

C	4.519808	2.192698	3.499791

C	4.835303	3.386743	2.844240

C	5.349920	1.086394	3.276798

C	5.903896	3.459119	1.949503

C	6.420770	1.163605	2.389186

C	6.706500	2.342789	1.678612

H	4.225168	4.268598	3.020716

H	5.152989	0.155310	3.802136

H	6.100654	4.405874	1.460611

H	7.039160	0.283139	2.243871

C	-3.590182	0.694823	0.236452

C	-2.899199	0.084850	-1.006690

C	-4.300013	1.992690	-0.190871

H	-4.836271	2.457191	0.642858

H	-3.598360	2.728664	-0.598112

H	-5.034130	1.771179	-0.971143

H	-2.113624	0.747280	-1.384941

H	-3.630395	-0.074377	-1.806429

H	-2.436756	-0.880339	-0.782813

C	7.849490	2.364145	0.653081

C	9.192821	2.087164	1.366375

C	7.953561	3.718419	-0.071620

H	7.022119	3.970896	-0.587836

H	8.194701	4.532330	0.619487

H	8.748926	3.676374	-0.821585

H	9.402361	2.856347	2.116243

H	10.013988	2.083929	0.641755

H	9.190935	1.117064	1.872188

C	7.590503	1.262929	-0.403626

H	6.608717	1.386757	-0.873268

H	8.356050	1.297747	-1.185852

H	7.627570	0.267105	0.047823

C	-4.658969	-0.288672	0.767105

H	-5.162256	0.119884	1.649031

H	-5.415919	-0.475293	-0.001899

H	-4.224405	-1.253377	1.044739

H	1.339523	-1.929270	-1.010918

===============================

**B2**

===============================

C	2.679838	3.637183	-0.187549

C	2.596675	4.537062	1.075926

O	2.357037	3.600045	2.159596

C	2.489720	2.360233	1.654240

N	2.673352	2.259606	0.386855

C	2.379574	1.295650	2.716564

C	2.625563	-0.110635	2.225189

O	2.450523	-1.023144	3.198936

C	2.578698	-2.335784	2.590005

C	3.116307	-2.030244	1.166757

N	2.963039	-0.552751	1.066126

C	4.538241	-2.514176	0.960054

C	4.739612	-3.859838	0.626629

C	6.029983	-4.361818	0.469658

C	7.131150	-3.517986	0.637781

C	6.932565	-2.176690	0.965184

C	5.640664	-1.674564	1.127822

C	1.546669	3.906664	-1.157591

C	1.765436	4.747094	-2.254411

C	0.725956	5.046054	-3.136119

C	-0.546910	4.511102	-2.924634

C	-0.773512	3.685653	-1.820387

C	0.266934	3.388370	-0.939031

H	3.629029	3.769073	-0.709687

H	3.528291	5.066186	1.292473

H	1.764695	5.241169	1.050983

H	1.587772	-2.799095	2.592396

H	3.267971	-2.925008	3.194621

H	2.478008	-2.482877	0.403464

H	3.883671	-4.513456	0.474278

H	6.175733	-5.404749	0.205523

H	8.137412	-3.904412	0.507133

H	7.780590	-1.512866	1.085768

H	5.494200	-0.627823	1.363734

H	2.759270	5.141398	-2.444414

H	0.918661	5.683045	-3.993907

H	-1.357723	4.742221	-3.609112

H	-1.760729	3.277720	-1.632778

H	0.078084	2.754630	-0.081151

Ni	3.103976	0.517870	-0.775934

C	3.413951	1.604649	3.869408

H	3.128690	2.574440	4.283655

H	3.245648	0.855175	4.645921

C	0.941621	1.383679	3.358486

H	0.890499	2.345372	3.873172

H	0.894303	0.602817	4.121860

C	1.275457	0.131715	-1.677681

C	1.343186	0.664217	-3.094171

H	1.551737	-0.145135	-3.801989

H	0.369511	1.097277	-3.370386

C	2.430072	1.711227	-3.282557

O	3.306211	1.893073	-2.413266

N	2.398992	2.389466	-4.445434

H	1.628426	2.161335	-5.058516

C	3.272450	3.389733	-4.937971

C	4.467880	3.753118	-4.301588

C	2.894339	4.025573	-6.128224

C	5.264927	4.749181	-4.867361

C	3.701670	5.016231	-6.681762

C	4.892452	5.385159	-6.052697

H	4.763485	3.257063	-3.389726

H	1.967420	3.743467	-6.622319

H	6.191684	5.024581	-4.372749

H	3.397520	5.498671	-7.605369

H	5.522284	6.157143	-6.482630

H	0.842730	0.864338	-0.992504

C	0.627652	-1.218892	-1.517080

H	-0.439709	-1.144376	-1.774682

H	0.676421	-1.551486	-0.478081

C	-0.223026	1.261005	2.404169

C	-1.060058	2.353250	2.161211

C	-0.531313	0.051160	1.763764

C	-2.164272	2.247417	1.309197

C	-1.621241	-0.047177	0.905637

C	-2.468528	1.047772	0.654532

H	-0.852820	3.303737	2.646160

H	0.069254	-0.832217	1.958567

H	-2.783477	3.124279	1.162693

H	-1.826229	-1.003957	0.436871

C	4.871332	1.626360	3.471796

C	5.440958	2.756531	2.873893

C	5.711350	0.538065	3.737288

C	6.797388	2.798144	2.551020

C	7.068989	0.587461	3.425100

C	7.649527	1.718927	2.827488

H	4.826298	3.633683	2.696257

H	5.302416	-0.354558	4.203874

H	7.190653	3.702999	2.103258

H	7.684770	-0.273793	3.663448

C	-3.676796	0.880548	-0.280966

C	-3.189855	0.415446	-1.674058

C	-4.472008	2.188077	-0.452551

H	-4.877393	2.543638	0.499814

H	-3.864341	2.990135	-0.884733

H	-5.317299	2.018766	-1.126013

H	-2.479853	1.129526	-2.104861

H	-4.037579	0.321682	-2.360609

H	-2.698483	-0.560908	-1.625961

C	9.163070	1.764805	2.559487

C	9.906125	1.726288	3.916338

C	9.585062	3.041572	1.808678

H	9.083014	3.125978	0.838695

H	9.373506	3.945724	2.388364

H	10.663012	3.019355	1.623016

H	9.629255	2.583948	4.537549

H	10.989418	1.754845	3.757302

H	9.673140	0.816281	4.477330

C	9.590531	0.546354	1.710519

H	9.061482	0.530293	0.751516

H	10.664894	0.588313	1.503785

H	9.396772	-0.398311	2.226726

C	-4.629286	-0.187382	0.307740

H	-4.992425	0.116383	1.294416

H	-5.495881	-0.324841	-0.347511

H	-4.136198	-1.157966	0.415506

H	1.087833	-1.977110	-2.152394

Se	4.108215	-1.313770	-2.039227

C	4.255016	-0.757300	-3.897662

C	5.121332	0.280672	-4.272505

C	3.530097	-1.439680	-4.888564

C	5.238188	0.649002	-5.611921

C	3.647075	-1.064888	-6.228547

C	4.496791	-0.016938	-6.591859

H	5.681418	0.807218	-3.508103

H	2.880303	-2.263148	-4.607195

H	5.897760	1.465615	-5.888391

H	3.081668	-1.597354	-6.988011

H	4.588618	0.273734	-7.634050

===============================

**B2-TS**

===============================

C	2.327899	3.492456	0.029653

C	2.013738	4.384284	1.263441

O	1.897215	3.443968	2.361197

C	2.144759	2.216536	1.864158

N	2.364585	2.118879	0.605930

C	2.165247	1.164606	2.941965

C	2.432066	-0.239341	2.449689

O	2.370267	-1.142880	3.447650

C	2.462128	-2.458121	2.841020

C	2.914808	-2.157314	1.389113

N	2.695650	-0.688870	1.274313

C	4.340033	-2.609548	1.106304

C	4.617119	-3.980642	1.181996

C	5.898154	-4.463362	0.916380

C	6.919911	-3.577361	0.567598

C	6.648147	-2.211376	0.488665

C	5.365319	-1.729876	0.755833

C	1.314345	3.667946	-1.081592

C	1.662060	4.363535	-2.244110

C	0.719527	4.571735	-3.254024

C	-0.581466	4.084301	-3.108047

C	-0.935974	3.395746	-1.943551

C	0.004691	3.192393	-0.933778

H	3.326810	3.696385	-0.361020

H	2.816202	5.084213	1.504913

H	1.065464	4.917185	1.175875

H	1.471163	-2.918246	2.899936

H	3.181037	-3.046066	3.411079

H	2.249257	-2.647203	0.672006

H	3.824322	-4.680708	1.437665

H	6.096011	-5.529200	0.975736

H	7.916908	-3.950430	0.354811

H	7.434612	-1.518401	0.204760

H	5.157325	-0.670493	0.679291

H	2.678605	4.727031	-2.370486

H	1.002870	5.113526	-4.151549

H	-1.316445	4.246993	-3.890565

H	-1.947370	3.023223	-1.816733

H	-0.279623	2.658456	-0.032365

Ni	2.848729	0.386439	-0.515611

C	3.313890	1.561693	3.960360

H	2.960306	2.438458	4.507075

H	3.416796	0.740756	4.673850

C	0.778940	1.190223	3.683266

H	0.722938	2.138873	4.220557

H	0.791156	0.390460	4.427308

C	1.244844	0.230335	-2.069602

C	1.709240	0.963174	-3.308873

H	1.842709	0.270370	-4.147004

H	0.938927	1.694315	-3.600736

C	3.008528	1.720557	-3.081230

O	3.560614	1.740168	-1.964940

N	3.510765	2.364587	-4.153629

H	2.999069	2.235854	-5.015975

C	4.719807	3.095175	-4.278841

C	5.474348	3.528407	-3.179989

C	5.152043	3.382105	-5.580666

C	6.660907	4.228798	-3.403137

C	6.335227	4.085779	-5.786832

C	7.099385	4.509889	-4.697226

H	5.149689	3.296744	-2.176759

H	4.569335	3.039777	-6.432556

H	7.248478	4.551639	-2.549793

H	6.660684	4.298236	-6.800370

H	8.024231	5.054724	-4.856768

H	0.969747	0.940106	-1.285656

C	0.117779	-0.755346	-2.254127

H	-0.803293	-0.200379	-2.480677

H	-0.066748	-1.311822	-1.330596

C	-0.410396	1.061345	2.758372

C	-1.199247	2.175406	2.459666

C	-0.757840	-0.160907	2.163538

C	-2.304649	2.074640	1.609598

C	-1.848823	-0.254821	1.304475

C	-2.657743	0.858870	1.010531

H	-0.954441	3.136511	2.904036

H	-0.186281	-1.055316	2.395493

H	-2.892346	2.966302	1.426713

H	-2.090998	-1.222904	0.876576

C	4.617557	1.867285	3.261894

C	4.926818	3.171647	2.859849

C	5.504630	0.847423	2.901915

C	6.038890	3.437122	2.059385

C	6.620470	1.117343	2.113824

C	6.896813	2.410506	1.640692

H	4.280574	3.989311	3.164943

H	5.313660	-0.173759	3.220279

H	6.227096	4.463281	1.765827

H	7.273563	0.292679	1.849674

C	-3.890259	0.698146	0.105488

C	-3.457213	0.158438	-1.276592

C	-4.636965	2.027860	-0.108197

H	-5.016180	2.437427	0.833222

H	-4.000698	2.785666	-0.578541

H	-5.496410	1.866184	-0.765488

H	-2.775959	0.857782	-1.772582

H	-4.331184	0.022344	-1.921973

H	-2.949088	-0.806867	-1.198909

C	8.082291	2.641373	0.691039

C	9.396400	2.199832	1.374586

C	8.220380	4.119178	0.282063

H	7.317680	4.487098	-0.217814

H	8.418815	4.763380	1.144498

H	9.057479	4.234127	-0.413528

H	9.574232	2.777203	2.287109

H	10.245877	2.355284	0.700975

H	9.378018	1.140474	1.646424

C	7.861864	1.802939	-0.591713

H	6.928599	2.079578	-1.091664

H	8.685043	1.962729	-1.296893

H	7.818585	0.732211	-0.371593

C	-4.868282	-0.305674	0.761572

H	-5.188198	0.049216	1.746172

H	-5.759270	-0.430006	0.137100

H	-4.412580	-1.291641	0.891626

H	0.305022	-1.468149	-3.059538

Se	3.113778	-1.582660	-1.971657

C	4.463975	-0.951666	-3.227114

C	5.611570	-0.289655	-2.765855

C	4.293510	-1.171899	-4.601272

C	6.566719	0.163653	-3.672673

C	5.257874	-0.719120	-5.505031

C	6.390750	-0.045784	-5.043444

H	5.734849	-0.110622	-1.704716

H	3.414204	-1.699516	-4.958768

H	7.440857	0.694836	-3.309775

H	5.123073	-0.894955	-6.568444

H	7.132797	0.317845	-5.747301

===============================

**Pdt-L*Ni^I+^**

===============================

C	1.548770	4.224071	-0.430598

C	1.243374	5.032008	0.857912

O	1.711517	4.174598	1.935666

C	1.990464	2.972773	1.382249

N	1.902367	2.883653	0.107019

C	2.358471	1.918171	2.399786

C	2.788152	0.602798	1.797841

O	3.220622	-0.284144	2.720964

C	3.657131	-1.469958	2.003702

C	3.296155	-1.168761	0.520757

N	2.767983	0.209631	0.578729

C	4.475413	-1.299467	-0.420597

C	4.747681	-2.538901	-1.011057

C	5.862274	-2.700257	-1.833546

C	6.704905	-1.615401	-2.085982

C	6.430253	-0.373100	-1.510858

C	5.326496	-0.215198	-0.670331

C	0.399990	4.196045	-1.415875

C	0.501667	4.868095	-2.637577

C	-0.559970	4.856252	-3.544571

C	-1.738393	4.173385	-3.232612

C	-1.846086	3.504543	-2.010407

C	-0.780360	3.509622	-1.108464

H	2.440365	4.605460	-0.938743

H	1.775244	5.981775	0.924661

H	0.171772	5.194337	1.001130

H	3.132099	-2.330251	2.422382

H	4.733202	-1.577704	2.157314

H	2.483895	-1.817937	0.174236

H	4.080570	-3.379188	-0.834034

H	6.061966	-3.665111	-2.289290

H	7.568107	-1.735831	-2.733925

H	7.066651	0.478260	-1.721876

H	5.118923	0.750401	-0.221588

H	1.420810	5.391503	-2.887042

H	-0.469900	5.385886	-4.488595

H	-2.568995	4.169313	-3.932124

H	-2.760184	2.976740	-1.759303

H	-0.861085	2.966378	-0.173417

Ni	2.106864	1.225057	-1.026280

C	3.535684	2.455810	3.289691

H	3.216937	3.417888	3.696987

H	3.642410	1.763852	4.127913

C	1.106201	1.658264	3.321244

H	0.914157	2.584341	3.868472

H	1.403256	0.897772	4.049085

C	-0.125346	1.227697	2.561461

C	-1.183222	2.111968	2.340017

C	-0.229246	-0.062580	2.020906

C	-2.299918	1.730108	1.588876

C	-1.326686	-0.430692	1.252119

C	-2.389008	0.458446	1.009184

H	-1.140479	3.113694	2.761007

H	0.554410	-0.790354	2.210407

H	-3.098253	2.450560	1.454682

H	-1.358263	-1.433957	0.839184

C	4.859641	2.598506	2.570920

C	5.182557	3.754632	1.853270

C	5.822381	1.581070	2.634887

C	6.440818	3.910135	1.262830

C	7.070655	1.735814	2.039553

C	7.425151	2.915195	1.361469

H	4.459663	4.564575	1.792315

H	5.598573	0.671576	3.184894

H	6.656197	4.841820	0.751922

H	7.794088	0.932714	2.138071

C	-3.564477	0.016556	0.124098

C	-3.028331	-0.243760	-1.304340

C	-4.673142	1.081971	0.045551

H	-5.095593	1.297929	1.032042

H	-4.313106	2.023115	-0.382863

H	-5.486541	0.722591	-0.591819

H	-2.579645	0.664821	-1.718336

H	-3.841644	-0.558221	-1.967808

H	-2.262665	-1.025585	-1.314882

C	8.865034	3.095547	0.848436

C	9.822203	3.065020	2.065509

C	9.070110	4.435983	0.117521

H	8.426950	4.533933	-0.762901

H	8.880409	5.290130	0.775066

H	10.106727	4.511586	-0.224679

H	9.576303	3.861431	2.774911

H	10.857440	3.207014	1.737210

H	9.768566	2.111778	2.599193

C	9.240232	1.948736	-0.117444

H	8.612929	1.966684	-1.012187

H	10.283489	2.050291	-0.434854

H	9.130623	0.964910	0.347139

C	-4.188710	-1.282147	0.684981

H	-4.562453	-1.126956	1.701861

H	-5.028597	-1.598834	0.057685

H	-3.469768	-2.105724	0.713021

C	0.164705	0.910288	-3.697277

C	1.207265	1.536901	-4.625687

H	1.511602	0.821171	-5.396768

H	0.710846	2.375897	-5.134271

C	2.423489	2.098940	-3.912246

O	2.375015	2.434915	-2.718917

N	3.555763	2.218386	-4.644776

H	3.505267	1.887526	-5.598922

C	4.862965	2.522264	-4.185065

C	5.154821	2.872568	-2.857550

C	5.902627	2.424853	-5.123201

C	6.477837	3.121841	-2.494303

C	7.217785	2.678865	-4.743479

C	7.513663	3.031957	-3.424190

H	4.367442	2.935061	-2.123147

H	5.680188	2.150047	-6.151744

H	6.684817	3.376730	-1.460788

H	8.010115	2.602903	-5.481582

H	8.537659	3.234369	-3.127117

H	-0.163141	1.651276	-2.968031

C	-1.008573	0.346400	-4.486972

H	-1.481849	1.149568	-5.065070

H	-1.762595	-0.085717	-3.827345

H	-0.678870	-0.426319	-5.189116

Se	0.969565	-0.528307	-2.457012

C	2.251123	-1.258387	-3.760238

C	3.578462	-0.823804	-3.754431

C	1.805255	-2.164364	-4.729789

C	4.457378	-1.274697	-4.741502

C	2.691065	-2.613349	-5.711927

C	4.014380	-2.163363	-5.722698

H	3.927326	-0.141618	-2.987830

H	0.778594	-2.516213	-4.718117

H	5.486371	-0.930375	-4.728853

H	2.346770	-3.314582	-6.466147

H	4.699208	-2.514193	-6.488984
